# Validation of Deep Learning-Based DFCNN in Extremely Large-Scale Virtual Screening and Application in Trypsin I Protease Inhibitor Discovery

**DOI:** 10.3389/fmolb.2022.872086

**Published:** 2022-06-01

**Authors:** Haiping Zhang, Xiao Lin, Yanjie Wei, Huiling Zhang, Linbu Liao, Hao Wu, Yi Pan, Xuli Wu

**Affiliations:** ^1^ Center for High Performance Computing, Joint Engineering Research Center for Health Big Data Intelligent Analysis Technology, Shenzhen Institutes of Advanced Technology, Chinese Academy of Sciences, Shenzhen, China; ^2^ School of Medicine, Shenzhen University, Shenzhen, China; ^3^ College of Software Technology, Zhejiang University, Hangzhou, China

**Keywords:** extremely large-scale virtual screening, deep learning, DFCNN, Trypsin I Protease, *de novo* drug screening

## Abstract

Computational methods with affordable computational resources are highly desirable for identifying active drug leads from millions of compounds. This requires a model that is both highly efficient and relatively accurate, which cannot be achieved by most of the current methods. In real virtual screening (VS) application scenarios, the desired method should perform much better in selecting active compounds by prediction than by random chance. Here, we systematically evaluate the performance of our previously developed DFCNN model in large-scale virtual screening, and the results show our method has approximately 22 times the success rate compared to the random chance on average with a score cutoff of 0.99. Of the 102 test cases, 10 cases have more than 98 times the success rate of a random guess. Interestingly, in three cases, the prediction success rate is 99 times that of a random guess by a score cutoff of 0.99. This indicates that in most situations after our extremely large-scale VS, the dataset can be reduced 20 to 100 times for the next step of virtual screening based on docking or MD simulation. Furthermore, we have employed an experimental method to verify our computational method by finding several activity inhibitors for Trypsin I Protease. In addition, we also show its proof-of-concept application in *de novo* drug screening. The results indicate the massive potential of this method in the first step of the real drug development workflow. Moreover, DFCNN only takes about 0.0000225s for one protein–compound prediction on average with 80 Intel CPU cores (2.00 GHz) and 60 GB RAM, which is at least tens of thousands of times faster than AutoDock Vina or Schrödinger high-throughput virtual screening. Additionally, an online webserver based on DFCNN for large-scale screening is available at http://cbblab.siat.ac.cn/DFCNN/index.php for the convenience of the users.

## Highlights

• The present work demonstrates that the DFCNN achieves high efficiency and relatively high accuracy. Here, we systematically evaluate the performance of our previously developed DFCNN model in large-scale virtual screening, and the results show our method has approximately 22 times the success rate compared to the random chance on average with a score cutoff of 0.99. Of the 102 test cases, 10 cases have more than 98 times the success rate of a random guess. Interestingly, in three cases, there is more than 1,000 times the success rate compared to the random guess by a score cutoff of 0.99. This indicates that in most situations after our extremely large-scale VS, the dataset can be reduced 20 to 100 times for the next step in virtual screening, usually based on docking or MD simulation.
• We have employed an experimental method to verify our computational method by finding several activity inhibitors for Trypsin I Protease. Among five experimentally tested compounds, STK573808 has the strongest binding affinity with the Ic 50 value of 1.16 mg/ml and Ka value of 1.86 × 10^6^ L mol^−1^. PB90939671, STK260654, and Z25746562 have a strong binding affinity, with an Ic 50 of 1.38, 1.42, and 2.98 mg/ml and Ka values of 1.87 × 10^3^, 3.60 × 10^4^, and 1.39 × 10^6^ L mol^−1^, respectively. To further check the possible interaction pattern between Trypsin I and each of the five compounds (Z25746562, STK260654, STK573808, PB90939671, and S763-0509), we have analyzed the MD simulation trajectory.
• We also show its proof-of-concept application in *de novo* drug screening. Interestingly, most of the novel compounds form much more hydrogen bonds and pi-related interactions than the inhibitors obtained from the ZINC database. This suggests DFCNN can also be applied to *de novo* drug screening when combined with a compound generative model, and it has the potential to discover new compounds with stronger inhibitory potency.

## Introduction

### The Current Status of Large-Scale Virtual Screening

Over the past several decades, computational-aided drug design-related technologies have widely been used in all stages of drug discovery (Yu and Mackerell, 2017). Among these technologies, large-scale virtual screening is routinely applied in the first stage of drug development (Kar and Roy, 2013). Many active compounds were successfully discovered with the help of the virtual screening method (Kitchen et al., 2004). However, the gap between the size of the database that the current method can process (around completing 10,000∼100,000 by typical computer with ∼10 CPU in weeks) and the availability of drug-like compounds (above 10,000,000 current purchasable structure) pose tremendous challenges, especially for those labs without supercomputing power. According to reports, the total number of drug-like molecules that are synthetically feasible theoretically was estimated to be around 10^30^ to 10^60^ (Popova et al., 2018). Among many virtual screening methods, the structure-based methods were the most popular (Lionta et al., 2014). However, the structure-based method requires correct receptor–ligand conformation before scoring, while finding the proper conformation through docking is often time-consuming (Cheng et al., 2012). Recently, an open-source drug discovery platform was developed for ultra-large virtual screens. However, it still requires supercomputers with tens of thousands of CPUs for screening a dataset with billions of compounds (Gorgulla et al., 2020).

Therefore, the accuracy of the binding conformation by docking still needs to be significantly improved (Wang et al., 2016). The ligand-based virtual screening approach is another alternative way to find drug candidates over an extensive chemical database quickly, for instance, LiSiCA (Lešnik et al., 2015) and LigandScout (Wolber and Langer, 2005). However, these methods usually require some known drugs that bind experimentally to the target, limiting their usage in novel drug development for a specific novel target. In addition, it does not directly include target information, making its accuracy questionable in many situations.

Many efforts for large-scale virtual screening have focused also on taking advantage of supercomputers and enhancing the efficacy of parallelization (Sánchez-Linares et al., 2012; Fang et al., 2016). Nevertheless, access to supercomputers can be difficult for most labs, and the cost is high.

### The Potential of Deep Learning in Large-Scale Virtual Screening

The docking technique is one of the most commonly used methods for large-scale virtual screening, and popular docking programs include Dock6 (Allen et al., 2015), AutoDock (Forli et al., 2016), AutoDock Vina (Trott and Olson, 2010), and Glide (Friesner et al., 2004). However, their efficacy is limited by the exhaustiveness of binding conformation searching. The accuracy of the traditional scoring function is questionable due to some implicit contributions such as the solvent effect and entropic effect that are hard to estimate (Ramírez and Caballero, 2018).

Due to the continuous increase of high-resolution protein–ligand complex in the PDB database (Goodsell et al., 2020), the accumulation of protein–ligand binding affinity data, and the rapid development of deep learning algorithms (Chen et al., 2018), it is now possible to build models that can detect the native-like complex efficiently and accurately. Many researchers focus on increasing the accuracy of structure-based methods (Back et al., 2019), which are usually obtained by docking. However, in large-scale virtual screening applications, the speed and accuracy should be balanced. Searching for correct protein–ligand binding conformations before the structure-based deep learning prediction would be too computationally expensive. Therefore, developing a deep learning method without the structure dependence would create models that are suitable for extremely large-scale virtual screening. DFCNN, our previously proposed model, has an advantage both in terms of both accuracy and speed (Zhang et al., 2019). Moreover, as one of the core components of a hybrid drug screening pipeline, DFCNN has already been successfully applied to discover novel inhibitors for some important targets such as RdRp (Zhang et al., 2020b) and TIPE2 (Zhang et al., 2021) in our previous research. However, the performance of DFCNN on different types of proteins is urgently needed to be systematically evaluated.

### High Precision Is Essential in Large-Scale Virtual Screening

Large-scale virtual screening requires some considerations that would be different from small-scale drug selection. Precision is one of the most critical performance indicators for large-scale virtual screening. Furthermore, the large-scale selection must ensure there are high percentages of activity compound (high precision) in the final selection pool; otherwise, the goal of virtual screening is not achieved. To sum up, high precision, while not so good accuracy is still acceptable for large-scale virtual screening.

### Our Current Work

In this work, we have systematically evaluated the effectiveness of the DFCNN model in identifying active and non-active compounds for 102 protein targets for the DUD.E dataset (Allen et al., 2015). It shows high precision for most of the cases. Furthermore, we have evaluated the model performance in extremely large-scale virtual screening using the top 10%, 20%, 30%, 40%, and 50% known active compound recall rate over around 10 million compounds. We also compare the ratio of TPR (true positive rate) by selecting compounds using 0.99 and 0.9 as score cutoff over the TPR of random selection rate. The result shows the huge potential of our model over most of the protein targets, except in three situations where the performance of membrane proteins is poor, especially with multiple pockets; proteins have a pocket binding with multiple compounds; and proteins have a pocket buried inside them with a relatively small cavity size. In addition, we utilize this DFCNN-based screening method to successfully find five novel active compounds for the target Trypsin I Protease. It should be noted that Trypsin I Protease has ranked third in terms of the ratio of predicted TPR over the TPR of random selection rate by using 0.99 as the cutoff. The result shows the potential of this method in large-scale virtual screening. Furthermore, such a systematic evaluation method has a generalized meaning for many other deep learning based protein–ligand models, which often make users uncertain about their scope of application. The poor performance of the DFCNN in the GPCR proteins also indicates that a GPCR-specific model should be trained based on the known GPCR-ligand dataset.

## Results

**FIGURE 1:**
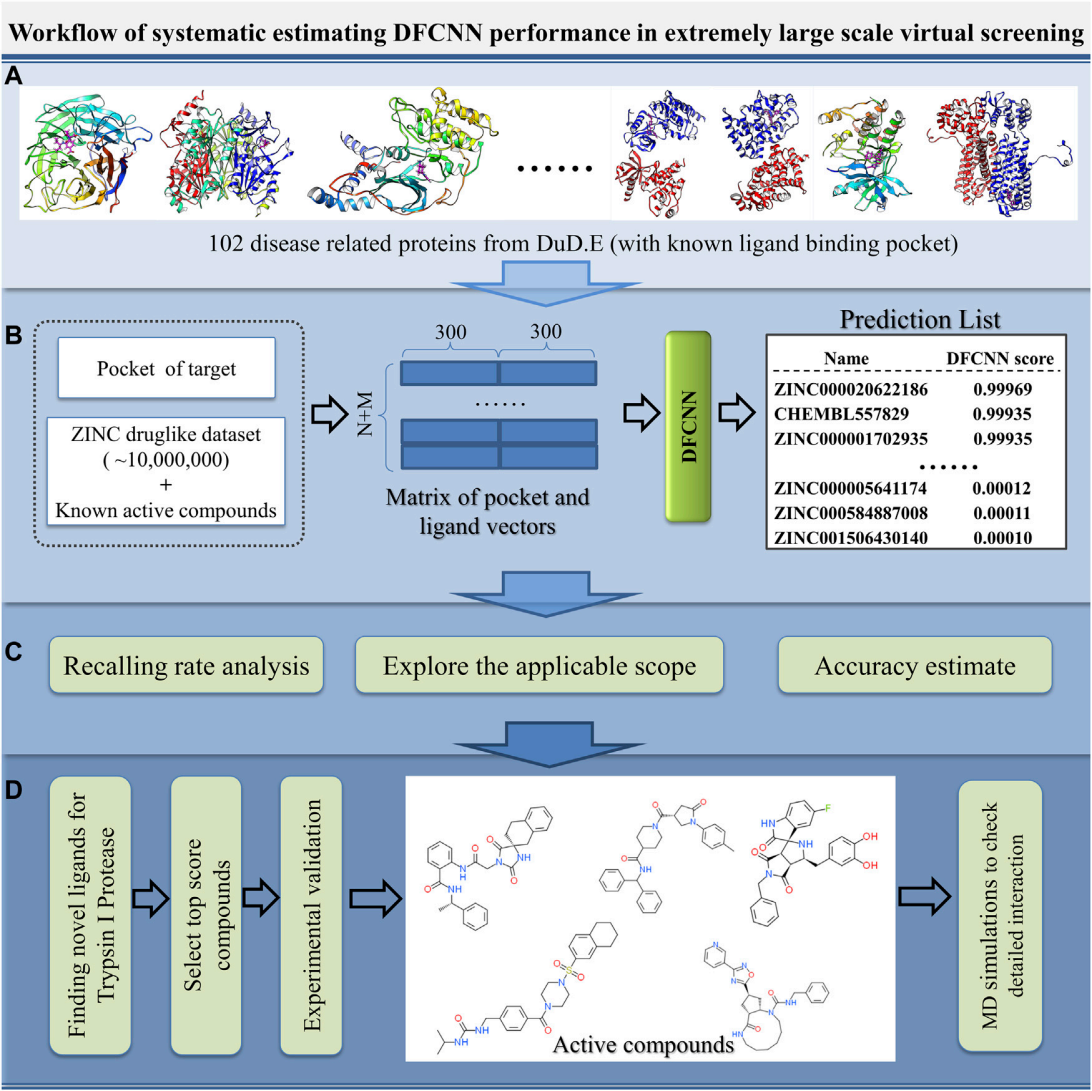
Schematic diagram of the systematic estimation of DFCNN performance in extremely large-scale virtual screening. **(A)** Collecting target protein-related data, **(B)** large-scale virtual screening against an extensive compound database (ZINC compounds plus known active compound) for each protein target, **(C)** doing various analyses based on the prediction, and **(D)** considering a good performance case (here, we use Trypsin I Protease) as a test example to find novel active compounds by combining the computational method with experimental validation.

With the increase in the number of experimentally determined protein–ligand complexes (and binding affinity data) and the rapid development of deep learning algorithms, deep learning in protein–ligand interaction prediction and drug virtual screening will become a new trend. However, the large-scale performance of deep learning-based methods on different proteins has to be systematically estimated urgently. Here, we systematically check the performance of DFCNN, one of our previously developed protein–ligand binding estimation models (Zhang et al., 2019), in large-scale virtual screening with the help of some known active compounds from the DUD.E dataset, shown in Figure 1. There are four steps. In Step A, we selected 102 diversified disease-related proteins from the DUD.E database, and each has known binding pockets and a set of known active compounds. In Step B, we predicted its pocket binding possibility over each compound in a large dataset (a ZINC dataset of ∼20,000,000 compounds plus known compounds of the targets). In Step C, we analyzed the recall rate. We checked which kinds of proteins are suitable for using this method based on the output from step B. We also estimated the accuracy of the prediction result of known active and inactive compounds. In Step D, we applied virtual screening novel to a target Trypsin I Protease, which was shown to have a good performance. In addition, MD simulation was used to detect the interaction details of these novel active compounds with the Trypsin I Protease.

### The Performance Test on the DUD.E Dataset With Known Inactive and Active Compounds

There are 101 cases in the DUD.E dataset that have provided both active and inactive compounds. We use these datasets to evaluate the performance of our model systematically. We use AUC, accuracy, TPR, precision, and MCC as performance indicators. The performance of 101 cases is shown in Supplementary Table S1. The positive data size and negative data size for each case were also shown. It can be noted that, for most cases, the precision was very high with an average value of 0.7736. It should also be noted that AUC and accuracy, and MCC are not performing in some cases. However, in a virtual screening application scenario, the high precision of the model is one of the most desirable. The TPR is also relatively high, with an average value of 0.6728. We selected the best performance cases with criteria of AUC ≥ 0.7, accuracy ≥ 0.7, TPR ≥ 0.7, precision ≥ 0.7, and MCC > 0 and show them in Table 1. We find that dopamine D3 receptor (3PBL), macrophage colony-stimulating factor receptor (3KRJ), and matrix metalloproteinase-13 (830C) also have demonstrated the highest precision. Still, the conclusion should be carefully made because of the minimal number of negative data compared to positive data. In terms of MCC value, farnesyl diphosphate synthase (1ZW5), acetylcholinesterase (1E66), and insulin-like growth factor I receptor (2OJ9) have the most reliable performance. However, the reliability of the test is limited due to the minimal size of the dataset, average of 225 positive and 90 negative data samples.

**TABLE 1:** Our model’s 20 best performance cases are from 101 cases with known active and inactive compounds. The criteria are AUC ≥ 0.7, accuracy ≥ 0.7, TPR ≥ 0.7, precision ≥ 0.7, and MCC > 0.

Name	AUC	Accuracy	TPR	Precision	MCC	Pos num	Neg num
3PBL	0.9479	0.9089	0.9083	0.9977	0.4347	480	14
3KRJ	0.7976	0.8304	0.8434	0.979	0.1108	166	5
830C	0.7593	0.9465	0.979	0.9655	0.2504	572	26
1LRU	0.815	0.7273	0.7353	0.9615	0.206	102	8
3CHP	0.7334	0.849	0.8713	0.9551	0.4302	171	21
2ZDT	0.8524	0.8189	0.8365	0.9355	0.5007	104	23
2ETR	0.7707	0.7826	0.81	0.931	0.3217	100	15
2OJ9	0.9048	0.8072	0.7703	0.9268	0.6177	148	75
2FSZ	0.8414	0.7789	0.782	0.9082	0.5018	367	126
2AYW	0.8323	0.8587	0.9198	0.9037	0.5584	449	117
1ZW5	0.9089	0.9018	1	0.8854	0.7244	85	27
2ICA	0.8126	0.7725	0.8333	0.8846	0.2883	138	29
2NNQ	0.8976	0.8413	0.9787	0.8364	0.5441	47	16
1SJ0	0.8385	0.79	0.9138	0.8216	0.4071	383	136
2HZI	0.7618	0.7669	0.8516	0.8158	0.4476	182	84
2E1W	0.8169	0.7556	0.8495	0.8061	0.4121	93	42
3KL6	0.7082	0.7938	0.9572	0.8056	0.355	537	176
1E66	0.8738	0.8074	0.8631	0.7667	0.6206	453	487
3EL8	0.7293	0.7374	0.9256	0.736	0.3912	524	287
Average	0.8212	0.8145	0.8752	0.8854	0.4275	268.5	90.2

We also carried out AutoDock Vina docking for the 19 proteins with their known active and inactive compounds from DUD.E for performance comparison, shown in Supplementary Table S2. DFCNN has a better average performance than AutoDock Vina (with median scores as cutoff) in all the used performance metrics. For instance, the average AUC is 0.8212 versus 0.6186, and the average MCC is 0.4275 versus 0.1819. The Schrödinger high-throughput virtual screening (HTVS) docking performance on the top five cases from Table 1 was used for comparison. Since our DFCNN performs well on these five targets, we want to further check whether DFCNN can perform better than Schrödinger on these five targets. If so, we can at least claim in some cases, DFCNN can even perform better than Schrödinger with a much faster speed. We admit that DFCNN cannot perform better in all the targets from Table 1 by the current version, but better performance on some kinds of protein with much faster speed would still demonstrate its usage in extremely large-scale virtual screening. As shown in Supplementary Table S3, it can be seen that Schrödinger has comparable precisions but worse performance on other calculated metrics. Notably, DFCNN has an extremely high speed compared with other methods, such as AutoDock Vina and Schrödinger docking. DFCNN could screen the 10 million drugs within 5 h using only a workstation with 80 Intel CPU cores (2.00 GHz) and 60 GB RAM. One protein–compound prediction only takes 0.0000225 s, while AutoDock Vina takes about 6 s with the same computational resource, which indicates DFCNN runs 266,667 times faster than AutoDock Vina. The Schrödinger’s HTVS docking takes about 1∼2 s for one protein–compound prediction on the 6 Intel(R) Core (3.00 GHz), which indicates DFCNN runs tens of thousands of times faster than Schrödinger’s HTVS.

### The Recall Rate Estimation in Large-Scale Virtual Screening

To systematically evaluate the model performance on the large-scale virtual screening, we have constructed a large drug-like compound dataset, with the size of 10,402,895 compounds. For each case, we predict the binding possibility of the protein pocket against those compounds in the dataset and their corresponding known active compounds. The recall rate of known active compounds in the top 10%, 20%, 30%, 40%, and 50% are shown in Supplementary Table S4. It can be seen that the top 50% of predictions have almost included all the active compounds for most cases, with an average recall rate of 0.7740. The top 40%, 30%, and 20% predictions all have recall rates higher than 0.5, shown in Supplementary Table S4. However, in a real application, we usually need a much smaller size of final compound candidates, so a recall rate of the top 10% would be more helpful information. For the top 10% prediction, the recalling rate is 0.3576, about 3.6 times higher than 0.1.

### The Estimation of Prediction–Random Ratio in Large-Scale Virtual Screening

We prefer using a certain score value as the cutoff in real applications. We tested the performance using score cutoffs of 0.9 and 0.99, respectively. The number of active compounds with scores more prominent than 0.99 or 0.9 is noted as $N_{0.99}$ or $N_{0.9}$ . The total number of active compounds for each protein was noted as $N_{total}$ . The prediction TPR ( $\text{P}\_{\text{tpr}}_{0.99}$ or $\text{P}\_{\text{tpr}}_{0.9}$ ) is defined by $N_{0.99}/N_{total}$ or $N_{0.9}/N_{total}$ . The total number of compounds with scores above 0.99 or 0.9 is defined as NN. The total compounds used in the test were defined as N_all. The random guess rate ( $random_{0.99}$ or $random_{0.9}$ ) is defined as NN/N_all . Finally, we describe the $P\_tpr_{0.99}/random_{0.99}$ as the prediction–random ratio with a cutoff of 0.99, and the $P\_tpr_{0.9}/random_{0.9}$ as the prediction–random ratio with a cutoff of 0.9. We use a prediction–random ratio with a cutoff of 0.99 and a prediction–random ratio with a cutoff of 0.9 as essential performance indicators in this work. The result is shown in Supplementary Table S5. It can be seen that using the cutoff of 0.99 and 0.9, most of the cases have a much higher predicted TPR value compared to random guess, with an average prediction–random ratio of 6.7321 and 3.1104, respectively.

**FIGURE 2:**
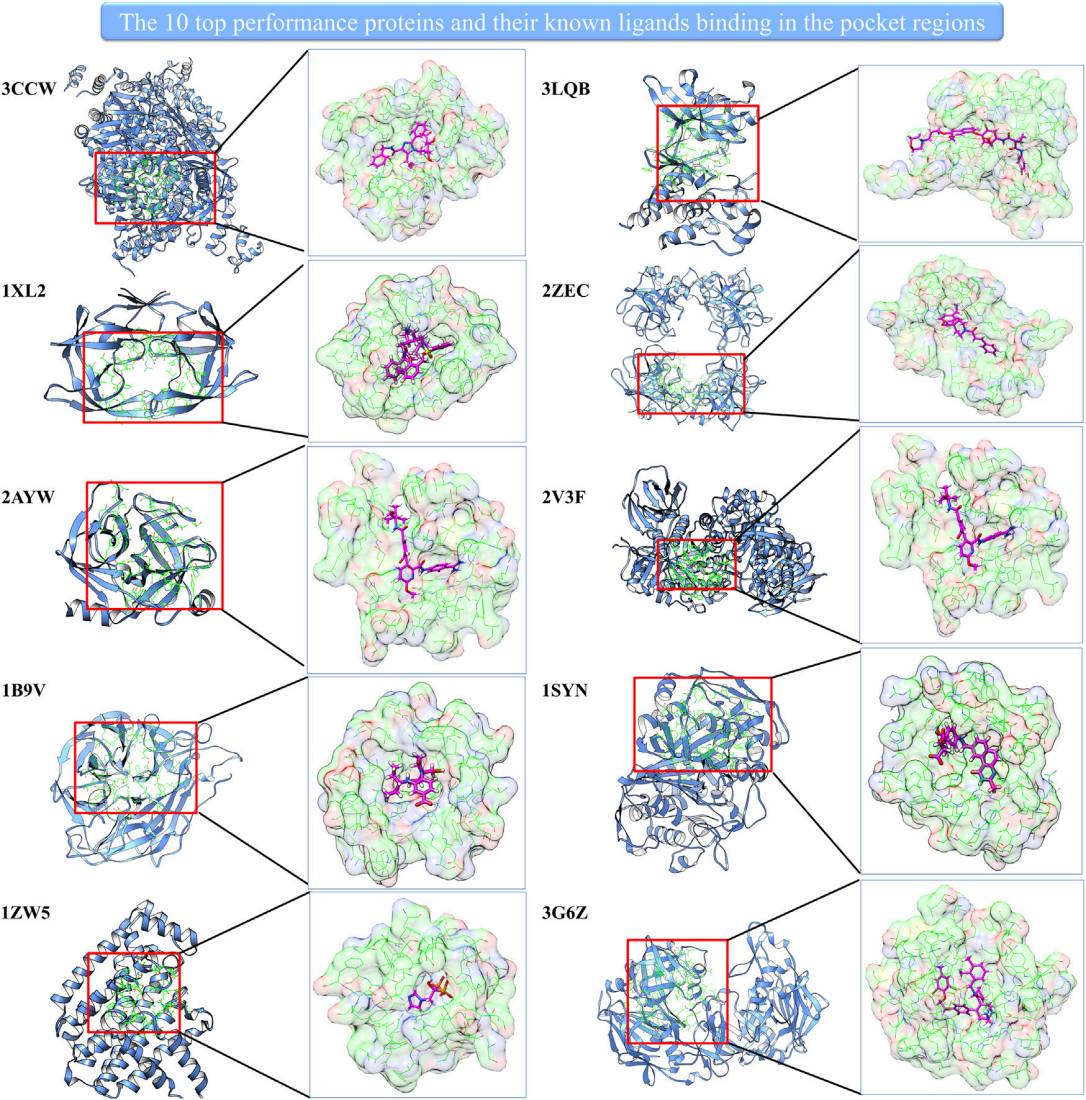
The 10 top performance proteins and their pocket regions with the known ligand.

Interestingly, the prediction–random ratio is hundreds or even thousands for some cases, which indicates our model can strongly enrich the active compounds into the high-score range for many famous therapeutic targets. We show 10 top performance proteins and their pocket regions with known ligands in Figure 2. It can be noted that all 10 proteins have large and easily accessed ligand binding pockets, and the pocket regions are relatively polar which favors hydrophilic interaction. This indicates our model can identify precise hydrophilic interactions, which has the potential to overcome the low specificity existing in traditional docking.

### Analysis of the Top 20 Performance Cases

The top 20 best performance cases are listed in Table 2. HMG-CoA reductase (HMDH) is the best performance target by our model, whose performance rate is ∼12,353 times that of random selection. HMDH’s function is related to the production of serum low-density lipoprotein cholesterol (LDL-c). Many of its inhibitors are effective drugs for the treatment of hypercholesterolemia. The second top performance target is human immunodeficiency virus type 1 protease (HIVPR), a well-known target for the treatment of HIV disease. Our method’s performance rate is 4,552 times that of a random selection of active compounds. The third best performance target is Trypsin I, a target for treating pancreatitis (trypsin inhibitors for the treatment of pancreatitis). Interestingly, our model performs well on several antihypertension-related targets, for instance, renin, an angiotensin-converting enzyme. Other top performance targets are also well known. Since our model can perform very well on these proteins, it is highly possible, among the compounds in the database that have a higher score than 0.99, there are many potential novel active compounds for those targets. The predicted compounds of these targets may greatly facilitate experimental groups to discover novel drugs. We have put this information on GitHub (https://github.com/haiping1010/potential_drug_compounds_of_20_target).

**TABLE 2:** The performance indicator of Ratio_0.99 ranks in the top 20 best performance cases.

PDB ID	Gene name	Protein name	Ratio_0.99
3CCW	HMDH	HMG-CoA reductase	12353
1XL2	HIVPR	Human immunodeficiency virus type 1 protease	4552
2AYW	TRY1	Trypsin I	1084
1B9V	NRAM	Neuraminidase	816.3
1ZW5	FPPS	Farnesyl diphosphate synthase	803.9
3LQ8	MET	Hepatocyte growth factor receptor	697.1
2ZEC	TRYB1	Tryptase beta-1	405
2V3F	GLCM	Beta-glucocerebrosidase	324
1SYN	TYSY	Thymidylate synthase	137.6
3G6Z	RENI	Renin	98.2
3KL6	FA10	Coagulation factor X	80.4
1LI4	SAHH	Adenosylhomocysteinase	79.3
3BKL	ACE	Angiotensin-converting enzyme	71
3E37	FNTA	Protein farnesyltransferase/geranylgeranyltransferase type I alpha subunit	68.4737
1SQT	UROK	Urokinase-type plasminogen activator	67.619
1NJS	PUR2	GAR transformylase	48.9796
1LRU	DEF	Peptide deformylase	46.6341
3F9M	HXK4	Hexokinase type IV	43.4
3EQH	MP2K1	Dual specificity mitogen-activated protein kinase kinase 1	40.5
2E1W	ADA	Adenosine deaminase	35.0972

### Analysis of the Top 10 Best Performance Cases

**FIGURE 3:**
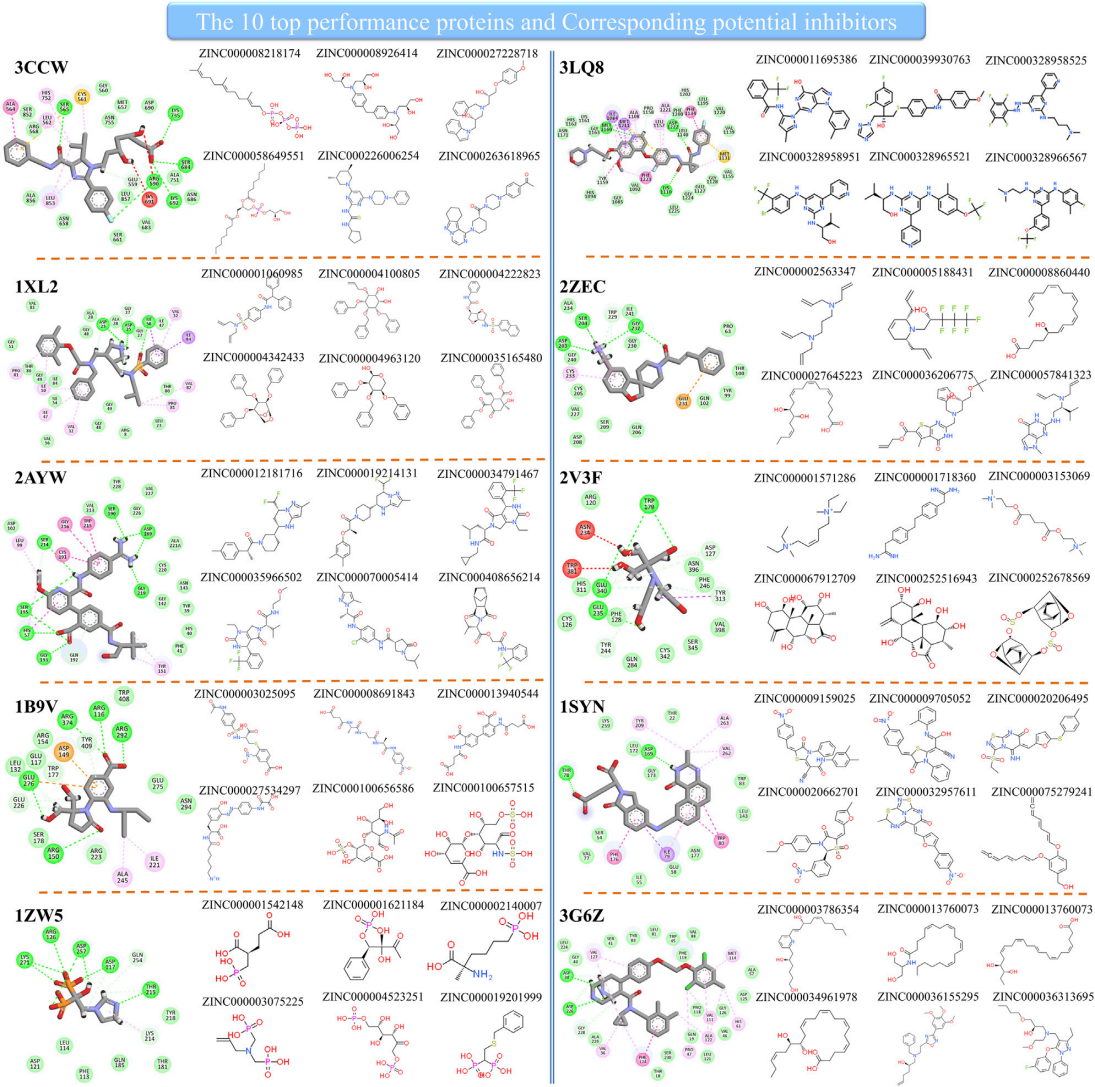
The top performance proteins and their corresponding potential inhibitors.

Since the method performed well on these 10 protein targets, the high-score compounds from the ZINC database are highly possible potential binders or inhibitors of these 10 targets. We clustered the high-score compounds into six groups for each of the targets shown in Figure 3. Clusfps (https://github.com/kaiwang0112006/clusfps), which depends on RDKit (Landrum, 2006), was used to complete the clustering with the algorithm of Murtagh (Murtagh and Contreras, 2012). Other experimental groups may gain clues from the structure of those compounds for developing drugs against these 10 therapeutic targets.

### Analysis of the Poor Performance Cases

**FIGURE 4:**
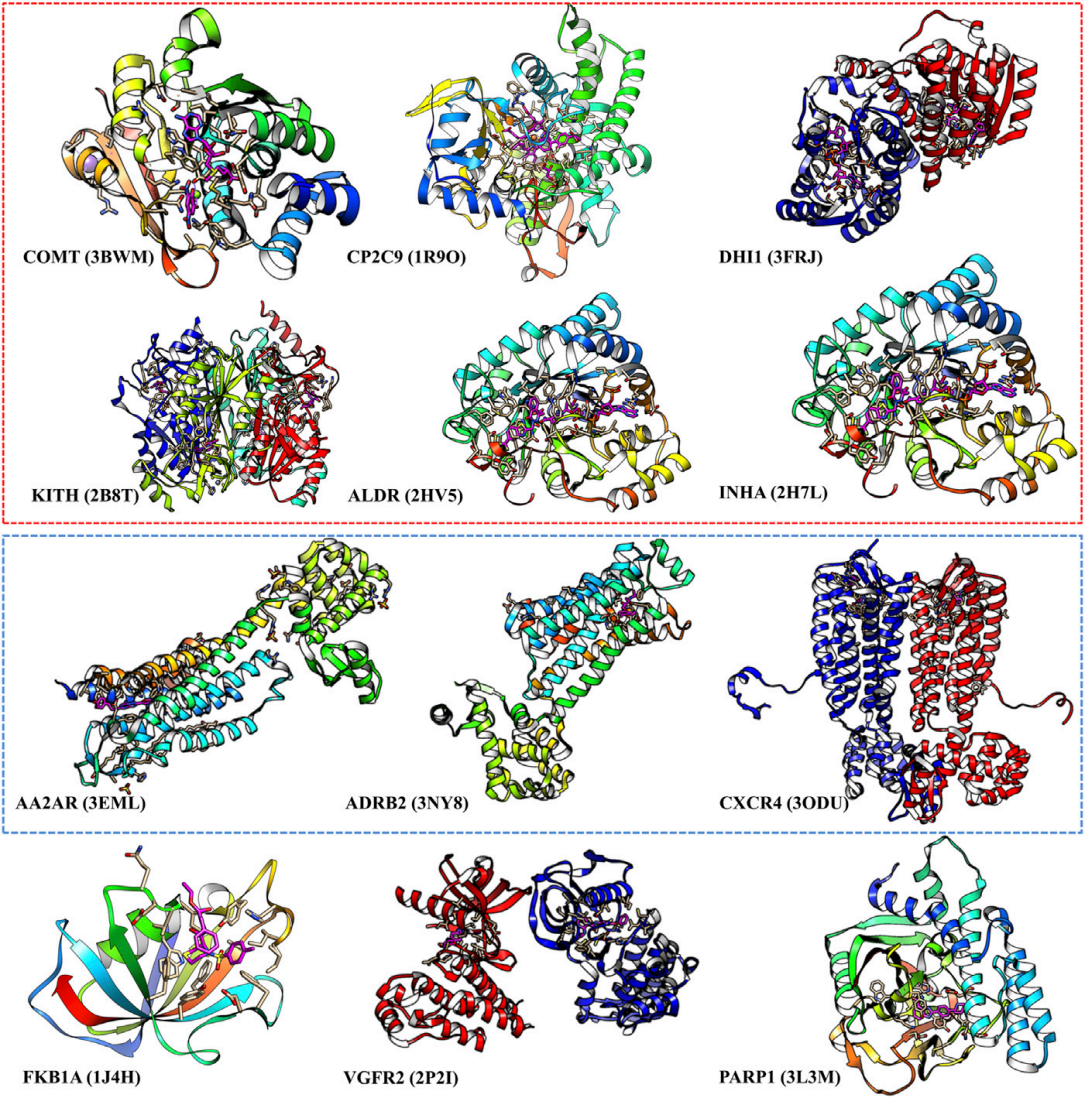
The poor performance proteins by the DFCNN. The gene names are annotated below, with the corresponding PDB ID shown in the bracket. The proteins within the red box have multiple ligands in one pocket, and the proteins within the green box are membrane proteins.

We select those cases with ratio values smaller than 1 with both score cutoffs of 0.9 and 0.99, as shown in Figure 4 and Supplementary Table S6. These poor performance cases can be categorized into three kinds of proteins: first, membrane proteins (including ADRB2, AA2AR, and CXCR4); second, cases where there are multiple ligands in the active pocket or ions in the active pockets (including CP2C9, DHI1 INHA, ALDR, and COMT); and third, cases where the pockets are deeply buried inside the protein, and the pocket space is relatively small, or the ligand–protein contact region is relatively small (including PARP1, FKB1A, KITH, and VGFR2). This indicates that on such cases, we should not use our model or add extra restraints during the ligand selection. This information also helps us design specific models instead of using DFCNN for GPCR-ligand prediction. It is indicated from Figure 4 that membrane proteins and proteins that have more than one ligand (or contain metal ions) are still too challenging by the current DFCNN method.

### Selecting Novel Compounds for Trypsin I (PDBid 2AYW)

Trypsin I is an important therapeutic target for hypercholesterolemia disease. We have some previous work on this target (Feng et al., 2018). In this work, our model performance was excellent in this target, ranked third in Table 2 and Supplementary Table S5 by a cutoff of score 0.99. The results strongly indicate some other novel active compounds in the prediction list (score higher or equal to 0.99). The 3D structure of all the compounds in the prediction list was downloaded from the ZINC database. After doing docking by AutoDock Vina, we selected that high-affinity one based on docking score and experience.

We manually checked the interaction pattern and binding affinity. Based on calculations and experience, we selected six compounds for the final experimental validation, shown in Supplementary Table S7.

### Experimental Validation of the Selected Compounds’ Binding With Trypsin I

**FIGURE 5:**
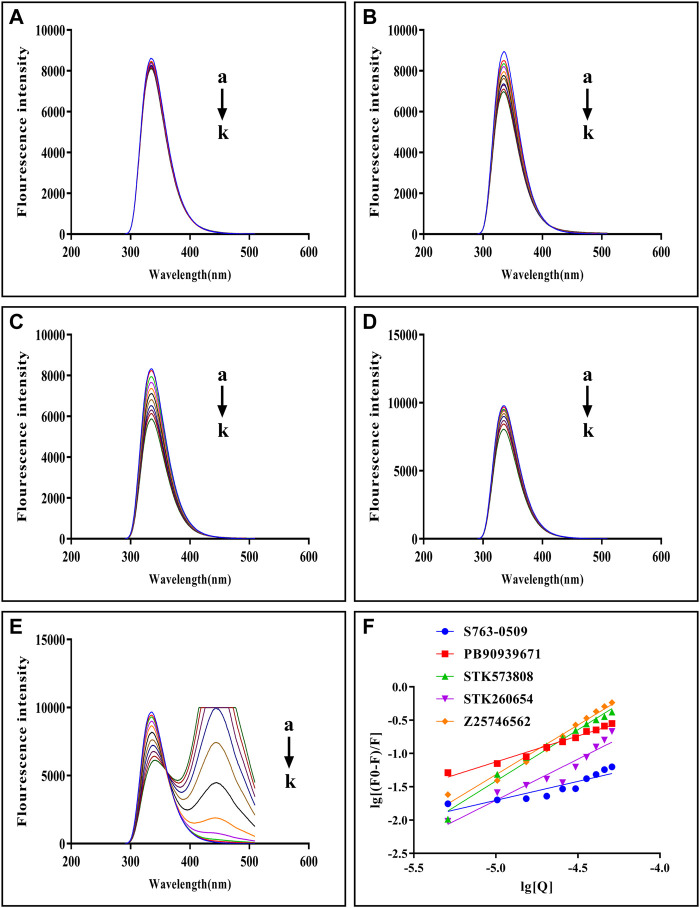
Fluorescence emission spectra of trypsin–S763-0509 **(A)**, trypsin–PB90939671 **(B)**, trypsin–STK573808 **(C)**, trypsin–STK260654 **(D),** and trypsin–Z25746562 **(E)** as well as double-log plots of the quenching effect of PPGs on trypsin fluorescence **(F)**. (a–k) The trypsin concentration was 1.0 × 10^–5^ M, and the compound concentrations were 0.0, 1.0, 2.0, 3.0, 4.0, 5.0, 6.0, 7.0, 8.0, 9.0, and 10.0 (×10^–4^ M).

The fluorescence quenching of the intrinsic Trp and Tyr fluorescence can be used to study the interactions between small-molecules and trypsin (Feng et al., 2018). As Figures 5A–E shows, with each compound’s gradual addition, the fluorescence intensity of trypsin decreased, thus implying that the compound might interact with trypsin Figure 5F shows double-log plots of the quenching effect of PPGs on trypsin fluorescence. The binding variables are shown in Supplementary Table S8. The lower value of IC50 or higher value of Ka indicates a stronger binding affinity. Hence, STK573808 has the strongest binding affinity with an IC 50 value of 1.16 mg/ml and a K_a_ value of 1.86 × 10^6^ L mol^−1^. The PB90939671, STK260654, and Z25746562 have relatively strong binding affinities, with an IC 50 of 1.38, 1.42, and 2.98 mg/ml and Ka values of 1.87× 10^3^, 3.60× 10^4^, and 1.39 × 10^6^ L mol^−1^, respectively. The S763-0509 has a weaker binding affinity with an IC 50 of 5.21 mg/ml and K_a_ of 6.77× 10^3^ L mol^−1^.

## Discussion

To explore the efficiency of DFCNN in virtual screening, we have checked the speed of our model in single prediction and large-scale virtual screening over 10402895 compounds. The virtual screening process took about 4 h of computational time under a Linux system with ∼40 CPU cores (1,000 MHz/core) for screening over each target. The virtual screening process took only 1.5 h of computational time with ∼ 1 CPU core (1,000 MHz/core) and GeForce RTX 2080 Ti for screening over each target. The high efficiency guarantees large-scale virtual screening over millions or even billions of compounds in a relatively short time and with accessible resources. The large scale turns back to increase the success rate of selecting desired drug candidates.

To make the DFCNN-based screening easy to access for most users, we have deployed an online webserver (http://cbblab.siat.ac.cn/DFCNN/index.php). Currently, it allows high-speed screening against Targetmol-Approved_Drug_Library, Targetmol-Natural_Compound_Library, and Targetmol-Bioactive_Compound_Library datasets for the known pocket of a given protein. In the future, we will progressively add new modules to make it able to screen larger datasets once we have more computational resources.

To explore the score distribution of the extensive virtual screening, we have plotted Supplementary Figure S1. Most compounds are at a low score range for these three top performance cases.

**FIGURE 6:**
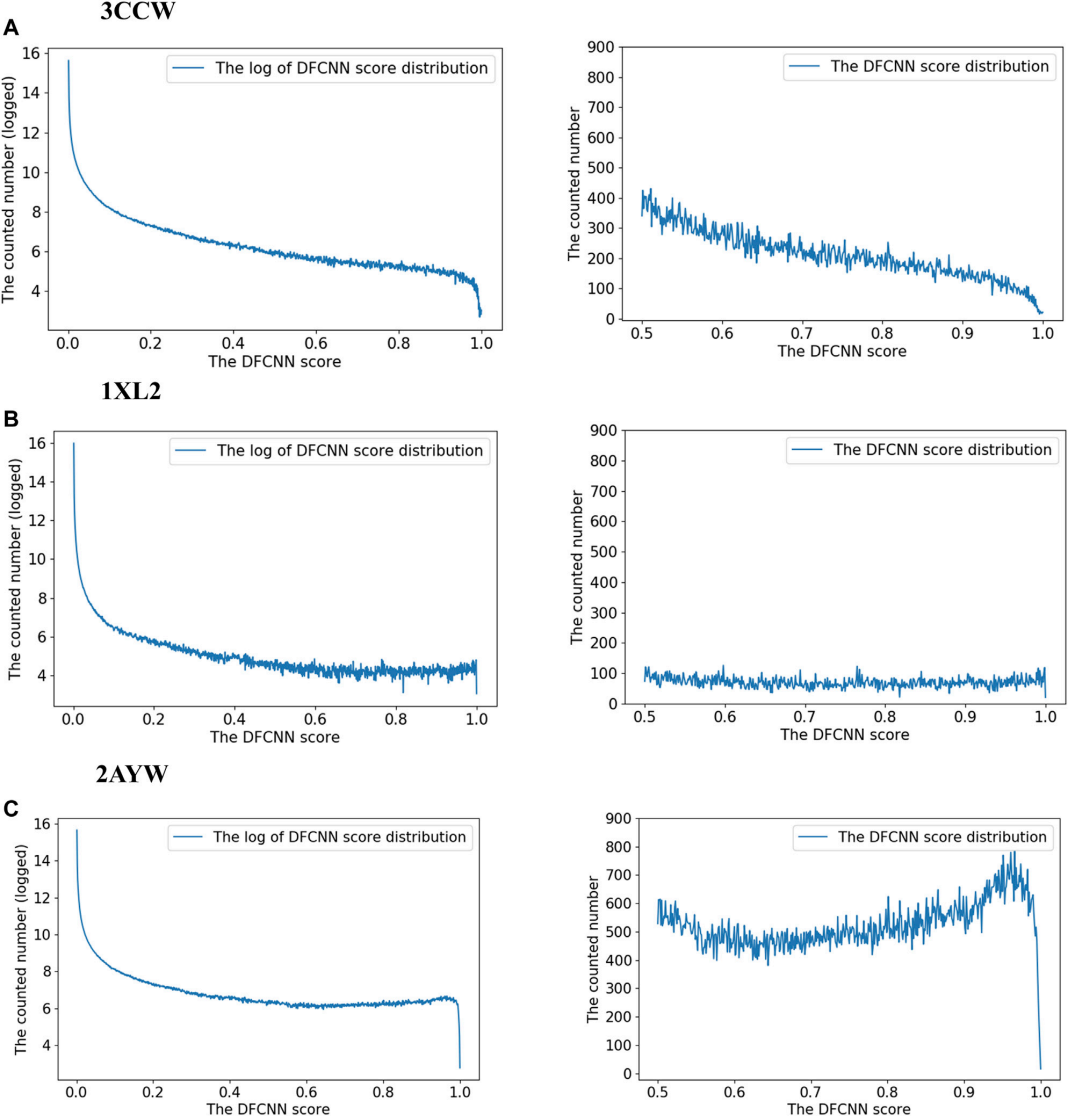
Analysis of the MD simulation result of Trypsin I with Z25746562 **(A)**, STK260654 **(B)**, STK573808 **(C)**, PB90939671 **(D)**, and S763-0509 **(E)**. The left panel shows the RMSD of Trypsin I and ligand (dark green and magenta) and indicates the number of hydrogen bonds between the protein and ligand. The middle panel shows the protein–ligand conformation of the last frame from the 100 ns MD simulation. The right panel shows the 2D diagram of the protein–ligand interaction from the last frame of MD simulation.

To check the interaction pattern between Trypsin I and each of the five compounds (Z25746562, STK260654, STK573808, PB90939671, S763-0509), we have analyzed the MD simulation trajectory, shown in Figure 6. Consistent with the experimental result, the MD simulation also shows that STK573808 has a stable binding with ligand RMSD around ∼0.4 nm. The last frame conformation has formed one hydrogen bond and four pi-related interactions. The PB90939671 and STK260654 also have relatively stable binding after 60 ns simulation. The Z25746562 and S763-0509 have relatively weaker binding stability with an RMSD value of around 0.6 nm. We noticed that S763-0509 forms a relatively more number of hydrogen bonds along 100 ns simulation, and it is known that hydrogen bonds may contribute to more binding specificity. By carefully examining the binding pose and interaction pattern of the five compounds (Figure 6, middle and right panel), we find that the detailed binding site and interaction residues in the pocket have relatively significant differences among the five ligands, indicating the different parts of the pocket may have the ability to bind relatively diversified compounds. This also suggests that our method can explore various binding compounds for one single pocket. This may help overcome the traditional drug design limitation, which often generates too similar compounds, and provides more opportunity to generate novel drugs for a given therapeutic target.

**FIGURE 7:**
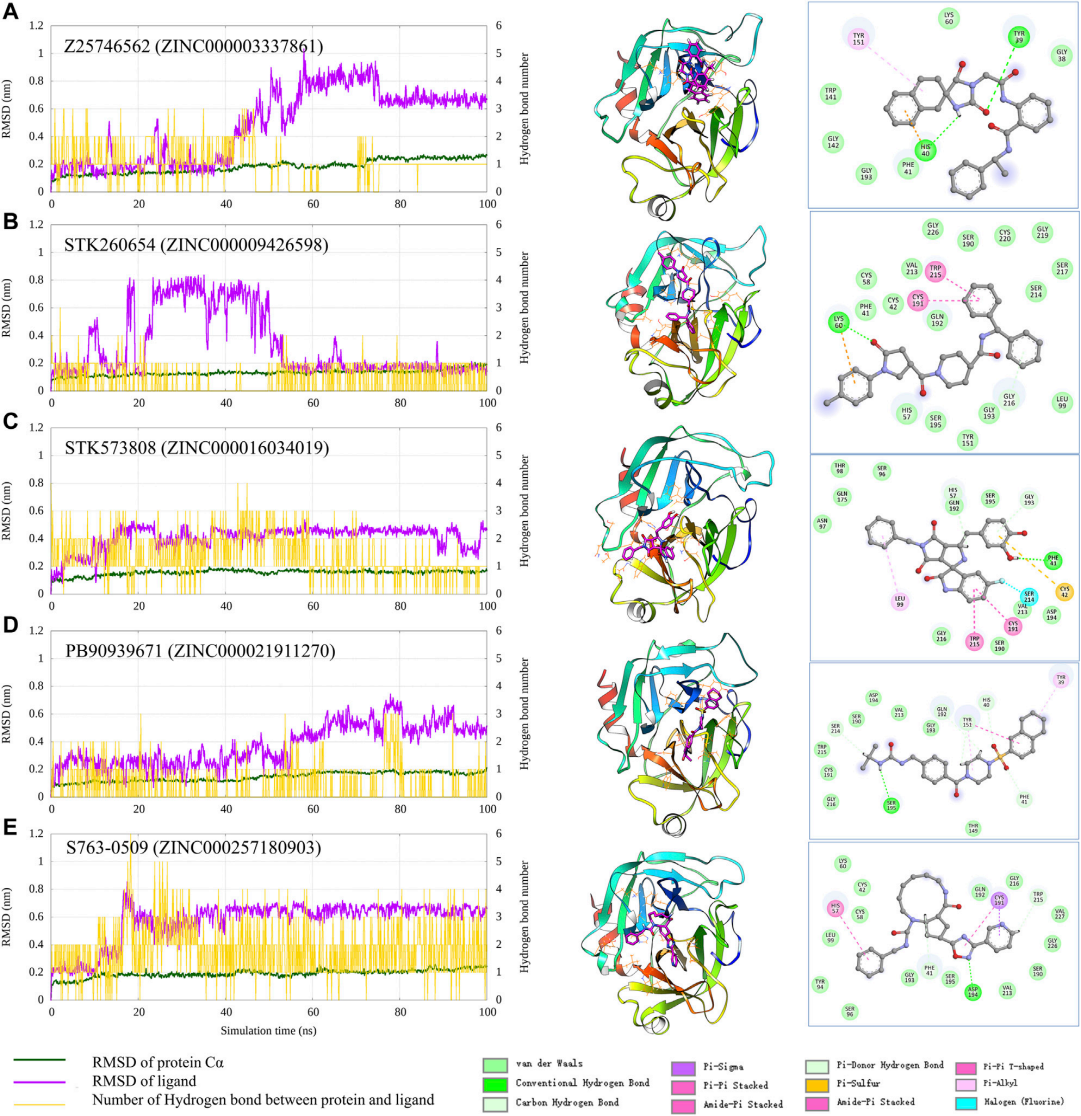
Representative structures of *de novo* candidates and their predicted interaction with the Trypsin I Protease.

To show the proof-of-concept application of DFCNN in *de novo* drug virtual screening, we carried out virtual screening by DFCNN over a *de novo* compound dataset, which was generated by a generative model LSTM_Chem with pre-trained weights. The detailed procedure of the compound’s generation process is shown in Supplementary Material Section S1. We only kept compounds that fulfilled Lipinski’s rule of five, and obtained 32 compounds with a DFCNN score ≥ 0.99 and AutoDock Vina score ≤ −8.5 kcal/mol. It should be noted that the number of totally generated unique compounds is 641582, the number of compounds that have a DFCNN score ≥ 0.99 is 2348, and among them, the number of compounds that fulfilled Lipinski’s rule of five is 317, with 32 out of 317 compounds ≤ −8.5 kcal/mol. We grouped the finally selected 32 compounds into six clusters, showing the representative compounds in Figure 7. We can also examine their predicted protein–ligand interaction pattern from the docked complexes. It can be noticed that most of the indicated compounds formed many strong interactions with Trypsin I Protease, including hydrogen bonds, pi-related interaction, and hydrophobic interaction, shown in Figure 7. Interestingly, most of the novel compounds form much more hydrogen bonds and pi-related interactions than the inhibitors obtained from the ZINC database in Figure 6. The representative compounds of Cluster 4 have six hydrogen bonds and four pi-related interactions. This suggests that DFCNN can also be applied to *de novo* drug screening when combined with a compound generative model and that it has the potential to discover new compounds with stronger inhibitory potency.

The traditional docking methods consider shape complementary but sometimes overestimate hydrophobic interaction, while our method emphasizes physical–chemical features and seems to have no such problems. This method is highly complementary to the traditional structure-based docking and MD simulation method. Assigning them in one pipeline greatly increases the success rate of first-stage drug development.

## Conclusion

The systematic evaluation demonstrates that the method can perform well in large-scale virtual screening. We evaluate the various performance indicators using the proteins’ known active and inactive datasets. Precision has a high score (average 0.766), which strongly supports the model suitable for virtual screening as precision is the most critical performance indicator in virtual screening. The performance of the very diverse protein types guides which type of protein is most suitable for our method. Analyzing several poor performance cases provides clues for further improving our model in some specific cases.

We have found a novel active compound for Trypsin I, which ranked third in the top best performance list. The successful finding of novel compounds in minimal test cases supports the effectiveness of our method in real applications. The method has performed impressively well in many other cases. For instance, the top 20 best performance proteins showed an average performance rate ∼1,093 times that of the random guess. It strongly suggests the candidate list, which contains compounds with DFCNN scores larger than 0.99, still potentially contains many activity compounds. The prediction list may be useful for other researchers who plan to design novel compounds for those targets. Our method is peer work that uses a limited number of CPU cores to screen whole 10 million compounds within several hours with high accuracy. The accuracy is guaranteed by incorporating protein pocket and ligand information as input, high-level deep learning architectures, and a comprehensive training dataset. This method shows strong potential in first-stage large-scale virtual screening. Since it is complementary to the traditional methods, such as docking, MD simulation, and structure-based protein–ligand binding estimators, it would be easier to identify novel drug leads by combining those methods in one pipeline.

## Methods

### The Model of Protein–Ligand Interaction Prediction

We adopt the trained model (DFCNN) from our previous work (Zhang et al., 2019; Zhang et al., 2020a; Zhang et al., 2020b; Zhang et al., 2021). Previously, the model was used for inverse target searching; here, we used it as a core module in extremely large-scale virtual screening. In addition, we have used DFCNN as a core model in a hybrid pipeline to help identify inhibitors for protein targets RdRp and TIPE2 in our previous works (Zhang et al., 2020b; Zhang et al., 2021). We have also applied DFCNN to do a virtual screening over the main protease of SARS-CoV-2 (Zhang et al., 2020a).

The deep learning-based method, DFCNN (Dense fully Connected Neural Network), has been developed for predicting the protein–drug binding probability (Zhang et al., 2019). DFCNN utilizes the concatenated molecular vector of protein pocket and ligand as input representation. The molecular vector is generated by Mol2vec (Jaeger et al., 2018), which is inspired by the word2vec model in natural language processing. The pocket was defined as residues within 1 nm of the known ligand. The DFCNN model was trained on a dataset extracted from the PDBbind database (Liu et al., 2015). The dataset’s negative samples were generated by cross-combination of proteins and ligands from PDBbind database, and positive data samples were taken from protein–ligand pairs in the experimental structure. DFCNN achieved an AUC value of around 0.9 for the independent testing set (Zhang et al., 2019). The model is about ∼100,000 times faster than AutoDock Vina in predicting the protein-ligand binding probability (range 0∼1) because it does not rely on the protein–drug complex conformation.

The architectural structure of the DFCNN model is illustrated in Supplementary Figure S2. We can see that DFCNN has 10 densely connected layers outputting 100 units simultaneously plus a standard fully connected layer outputting one unit as the final output of this model. Specifically, a densely connected layer means a layer taking all outputs of its preceding layers as its input that could remarkably solve the gradient vanishing problem. The CNN comprises two convolution blocks consisting of two 1D convolutional layers, a max pool layer severally and a dropout layer, a flattening layer, a dense layer outputting 256 units, and a dropout layer, and finally, a dense layer outputting a single unit. Rates for dropout layers are all 0.25. All of the convolutional and dense layers employed the ReLU activation function except the output layers, which employed the sigmoid activation function. Input for the two networks has been normalized to make its mean and standard deviation be 0 and 1 separately. The RMSprop optimizer was used to minimize the binary cross entropy of CNN and the Adam optimizer was used to minimize the binary cross entropy of DFCNN. The model architecture, the training data preparation, and the training and testing procedures can be found in our previous work in detail (Zhang et al., 2019; Zhang et al., 2020a; Zhang et al., 2020b; Zhang et al., 2021).

### Drug-Like Compound Database for Virtual Screening

We have downloaded the 2D compound files in smi format from the ZINC15 database (Sterling and Irwin, 2015). The compounds were selected based on the following criteria: drug-like compound, pH 7, pursuit status was in stock, having available 3D structures, and can be processed by RDKIT (Landrum, 2011). We collected 10,402,895 compounds as the final virtual screening database (VS_DB).

### Building the Virtual Screening Pipeline

The virtual screening pipeline is shown in Figure 1. The protein pocket was extracted and converted into a 300-dimension vector by the mol2vec tool (Jaeger et al., 2018); the atoms around 1 nm within the known ligand were defined as the protein pocket in this work. All the ligands in the VS_DB were converted into a 300-dimension vector by the mol2vec tool (Zhang et al., 2019). A homemade python script was then used to combine the protein vector with each ligand vector in the VS_DB as later input vectors. The model was used to evaluate the binding possibility of each protein–ligand in the VS_DB by learning the input vectors.

### Systematically Evaluating the Model Performance on the DUD.E Dataset

We selected 101 DUD.E protein datasets, which both have known activity compounds and known inactivity compounds. We use AUC, accuracy, TPR, MCC, and precision as performance metrics. We also test the performance of AutoDock Vina in the same dataset for comparison.

### Systematically Validating the Efficacy of the Model on Large-Scale Virtual Screening

We use the 102 protein targets from the DUD.E dataset to do the validation. We have done a virtual screening for these 102 proteins against the virtual screening database. We also conducted virtual screening for the 102 protein targets with their known active ligands (we chose the one from CHEMPB here). The known active ligands were downloaded from the DUD.E webserver. An average of ∼225 known activity compounds and ∼90 known inactive compounds are for one target.

After the virtual screening, the score was ranked. The percentage of activity compounds within the top 10%, 20%, 30%, 40%, and 50% of predicted scores for each case was calculated, respectively, and taken as performance indicators.

We also checked how many active compounds have possibility scores higher than 0.99 and 0.9. We also evaluated the total number of compounds with possibility scores higher than 0.99 and 0.9. The number of active compounds with scores higher than 0.99 or 0.9 is noted as $N_{0.99}$ or $N_{0.9}$ . The total number of active compounds for each protein was noted as $N_{total}$ . The prediction TPR ( $\text{P}\_{\text{tpr}}_{0.99}$ or $\text{P}\_{\text{tpr}}_{0.9}$ ) is defined by $N_{0.99}/N_{total}$ or $N_{0.9}/N_{total}$ . The total number of compounds with a score above 0.99 or 0.9 is defined as $NN_{0.99}$ or $NN_{0.9}$ . The total number of compounds used in the test is defined as N_all. The random guess rate ( $random_{0.99}$ or $random_{0.9}$ ) is defined as $NN_{0.99}/N\_all$ or $NN_{0.9}/N\_all$ . Finally, we describe the $P\_tpr_{0.99}/random_{0.99}$ as the prediction–random ratio with a cutoff of 0.99 (Ratio_0.99), and the $P\_tpr_{0.9}/random_{0.9}$ as the prediction–random ratio with a cutoff of 0.9 (Ratio_0.9).

$$Ratio\_0.99=P_{tpr0.99}/random_{0.99}=\left(N_{0.99}/N_{total}\right)/\left(NN_{0.99}/N\_all\right),$$ (1)

$$Ratio\_0.99=\left(N_{0.99}/NN_{0.99}\right)/\left(N_{total}/N\_all\right),$$ (2)

$$Ratio\_0.9=P_{tpr0.9}/random_{0.9}=\left(N_{0.9}/N_{total}\right)/\left(NN_{0.9}/N\_all\right),$$ (3)

$$Ratio\_0.9=\left(N_{0.9}/NN_{0.9}\right)/\left(N_{total}/N\_all\right).$$ (4)

The Ratio_0.99 can be expressed by either Eqs 1 or 2, and the $Ratio_{0.9}$ can be expressed in either Eqs 3 or 4. The Eqs 2, 4 can be easily understood in terms of statistics.

### Docking for the Top 20 Performance Targets

We have selected 20 targets that show the best performance in the prediction–random ratio with a cutoff of 0.99 ( $P\_tpr_{0.99}/random_{0.99}$ ). For all compounds in VS_DB that have scored better than 0.99, we downloaded their 3D compounds from the ZINC database and performed traditional AutoDock Vina docking. We docked all the compounds that had predicted DFCNN scores above 0.99 to the Trypsin I protein known pocket by AutoDock Vina. The scripts named “prepare_receptor4.py” and “prepare_ligand4.py” from AutoDockTools were used for preparing AutoDock Vina input files, respectively (Morris et al., 2009). The pocket size was set to include the active binding site, using a box size of 25, 25, 25 Å. The docking center is the center of the known protein pocket, composed of residues within 1 nm of the known ligand. For each protein–ligand docking, we generate a maximum of 20 conformations. The top 40 docking conformations in terms of scores were selected. We use structure-based docking here because it has considered spatial information, which is highly complementary to our method.

### Identifying Novel Active Compounds of Trypsin I by Our Pipeline for Experimental Validation

We selected potential inhibitors for Trypsin I based on the DFCNN score, docking score, and visional observation of docking conformation. Nine compounds were chosen for final experimental validation. The experimental protocol to evaluate the binding strength and activity is described in Supplementary Material Section S2. We also carried out molecular dynamics simulations to check the ligand-binding mode and detailed binding pattern. The detailed procedures are illustrated in Supplementary Material Section S3.

## Data Availability

The original contributions presented in the study are included in the article/Supplementary Material, further inquiries can be directed to the corresponding authors.
