# Supplementary material for: Validation of Deep Learning-Based DFCNN in Extremely Large-Scale Virtual Screening and Application in Trypsin I Protease Inhibitor Discovery
Source: Front Mol Biosci. 2022 Jun 1;9:872086. doi: 10.3389/fmolb.2022.872086 (PMC9200220; doi:10.3389/fmolb.2022.872086)
Supplement: Supplementary file 1 [file DataSheet1.docx]

**Supplementary material section 1. The procedure of generated de novo compound dataset and the following virtual screening.**

We directly used the pre-trained model from a publicly accessible code(https://github.com/mattroconnor/deep_learning_coronavirus_cure), which is contributed by Matt O'Connor in an open-source Coronavirus Drug Discovery Competition. The generative model structure is from LSTM_Chem (Generative Recurrent Networks for De Novo Drug Design) (<https://github.com/topazape/LSTM_Chem>). We set the parameters to generate 1 million compounds and removed those redundant ones, finally obtained 641582 compounds. With the dataset, we have done the virtual screening by DFCNN with the same setting as previously described. After that, we only select the compounds that satisfied Lipinski's rule of five and have DFCNN score >=0.99 to do the docking. The docking settings are the same as in our previous paper (Zhang et al., 2020). Finally, we obtain 32 selected compounds that have a DFCNN score >= 0.99 and Autodock Vina score <= -8.5 kcal/mol. Clusfps (https://github.com/kaiwang0112006/clusfps), which depends on RDKit (Landrum, 2006) was used to cluster the 32 compounds into 6 groups with the algorithm of Murtagh (Murtagh and Contreras, 2012).

**Supplementary material section 2.** **Experimental protocol to evaluate the binding strength and activity.**

*Materials*

Bovine trypsin (2500 U/mg) was from Sigma-Aldrich Co. (St. Louis, MO, USA).

*Determination of trypsin activity*

Trypsin activity was determined with BAPNA as the substrate, as described previously(Feng et al., 2018).

*Determination of fluorescence spectroscopy*

Fluorescence spectra was measured in a temperature-controlled environment (37 °C) on a Hitachi-850 spectrofluorometer (Hitachi Co., Tokyo, Japan) equipped with 1.0-cm quartz cells. A 2-mL sample of trypsin solution (1 μM) was placed into the cell. Different volumes (μL) of compound solution (1 mM) were added to obtain molar ratios of compound:trypsin of 0, 1, 2, 3, 4, 5, 6, 7, 8, 9, and 10. All samples were analyzed in triplicate. According to previous reports(Feng et al., 2018), the intensity at 340 nm was used to calculate the number of binding sites per enzyme (n) and the binding constant (Ka).

Fluorescence emission spectra were recorded at an excitation of 280 nm over a wavelength range of 300–450 nm, and the excitation and emission bandwidths were 5 nm. A fixed volume (3.0 mL) of 1.0 × 10^−6^ M trypsin was titrated by successively adding each different PPG (acteoside, lipedosideA-I, syringalide A 3ʹ-α-L-rhamnopyranoside, and osmanthuside B) solution (1 × 10^-3^M). According to a previous report(Xu et al., 2019), the intensity at 340 nm was used to calculate the number of binding sites per enzyme (n) and the binding constant (*Ka*).

**Supplementary material section 3: Molecular dynamics simulation to check the ligand binding mode and detailed binding pattern**

GROMACS 4.6 software was used to perform atomistic molecular dynamics simulations with an all-atom AMBER99SB force field (Hornak and Simmerling, 2003; Hess et al., 2008) for the top five protein-ligand complexes sorted based on docking scores. ACPYPE, which depends on Antechamber was used to generate ligand structural topology(Wang et al., 2006; Sousa Da Silva and Vranken, 2012). The protein-ligand complex system was solvated in a cubic box (size determined by keeping the closest protein atom to the box boundary at 1 nm) of TIP3P (transferable intermolecular potential with 3 points) water(Jorgensen et al., 1983) using minimization with the steepest descent algorithm. The net charge of the system was neutralized by adding counter ions. Charge interactions (electrostatic) were computed by the Particle Mesh Ewald method(Darden et al., 1993). After refining the complex by energy minimization, a 1-ns isothermal-isovolumetric ensemble simulation was carried out to equilibrate the water box with a force constant of 1000 kJ/(mol∙Å) in x, y, and z dimensions. A 1-ns NpT (isobaric-isothermic) ensemble simulation was used to equilibrate the water box in 1 atm pressure with a force constant of 1000 kJ/(mol∙Å) in each dimension. Subsequently, another 100 ns NpT ensemble MD simulation was performed for production simulation with a fixed temperature of 308 K at 1 atm pressure. Anisotropic diagonal position scaling with a time step interval of 0.002 ps was employed to maintain constant pressure during MD simulations. Additionally, the Berendsen algorithm and Lennard-Jones cut-off value were fixed at 0.2 constant and 9 Å, respectively. Hydrogen bond formation was analyzed with the g_bond tool in Gromacs program modules. Analysis and plotting of results were carried out by using VMD and other standard inbuilt tools in Gromacs software(Humphrey et al., 1996). The trajectory files are saved appropriately for further computations.

**Supplementary Figures:**


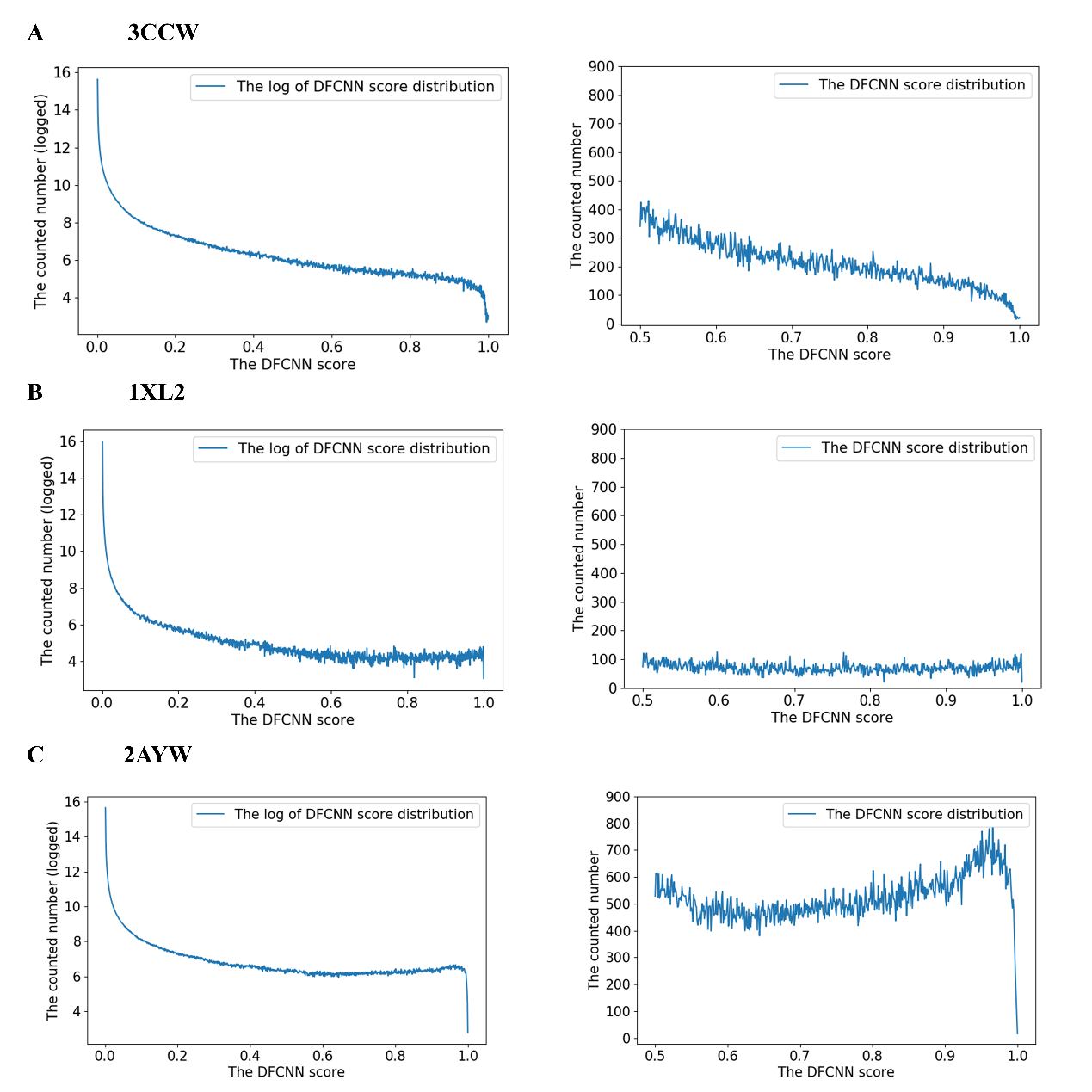
**Figure S1. The DFCNN score distribution of the top performance 3 cases.** The left panel shows the score distribution from 0 to 1, with logged counted numbers for better observation due to the large number of compounds are in the low score region. The left panel shows the score distribution from 0.5 to 1, the counted number shown on the Y-axis.


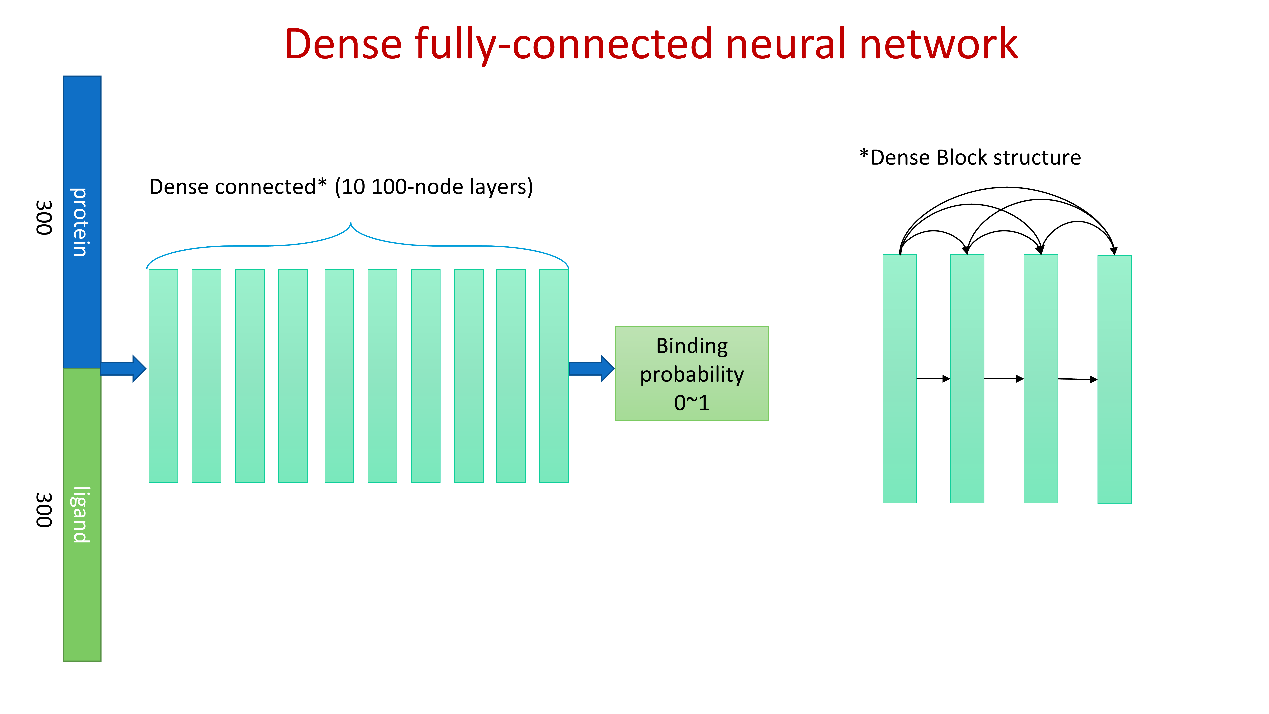


**Figure S2.** The architecture of Dense Fully Connected Neural Network (DFCNN).

**Supplementary Tables:**

**Table S1. The performance of our model on the 101 cases with known active and inactive compounds.**

| Name | AUC | Accuracy | TPR | Precision | MCC | Pos_num | Neg_num |
| --- | --- | --- | --- | --- | --- | --- | --- |
| 1LI4 | 0.6222 | 0.2647 | 0.2063 | 1 | 0.137 | 63 | 5 |
| 3BKL | 0.7226 | 0.2344 | 0.0851 | 1 | 0.1223 | 282 | 55 |
| 3BWM | 0.6098 | 0.0952 | 0.0732 | 1 | 0.0433 | 41 | 1 |
| 3L3M | 0.8617 | 0.4673 | 0.4547 | 1 | 0.1374 | 508 | 12 |
| 3PBL | 0.9479 | 0.9089 | 0.9083 | 0.9977 | 0.4347 | 480 | 14 |
| 3KBA | 0.2014 | 0.7585 | 0.7611 | 0.9955 | -0.0327 | 293 | 1 |
| 3KRJ | 0.7976 | 0.8304 | 0.8434 | 0.979 | 0.1108 | 166 | 5 |
| 3BQD | 0.5536 | 0.8636 | 0.8798 | 0.9784 | 0.0212 | 258 | 6 |
| 2AA2 | 0.1117 | 0.8542 | 0.8723 | 0.9762 | -0.0551 | 94 | 2 |
| 3CCW | 0.6529 | 0.573 | 0.5706 | 0.97 | 0.0817 | 170 | 8 |
| 830C | 0.7593 | 0.9465 | 0.979 | 0.9655 | 0.2504 | 572 | 26 |
| 1XL2 | 0.8386 | 0.7025 | 0.6735 | 0.9652 | 0.3929 | 536 | 96 |
| 1LRU | 0.815 | 0.7273 | 0.7353 | 0.9615 | 0.206 | 102 | 8 |
| 3HMM | 0.6144 | 0.9357 | 0.9774 | 0.9559 | 0.1574 | 133 | 7 |
| 3CHP | 0.7334 | 0.849 | 0.8713 | 0.9551 | 0.4302 | 171 | 21 |
| 3EQH | 0.6928 | 0.6917 | 0.6942 | 0.9545 | 0.2185 | 121 | 12 |
| 3LPB | 0.5047 | 0.8407 | 0.8785 | 0.9495 | 0.0307 | 107 | 6 |
| 1MV9 | 0.4918 | 0.7319 | 0.7634 | 0.9434 | -0.0488 | 131 | 7 |
| 2AM9 | 0.6243 | 0.8229 | 0.8625 | 0.9431 | 0.0883 | 269 | 19 |
| 3HL5 | 0.4471 | 0.8692 | 0.92 | 0.9388 | 0.056 | 100 | 7 |
| 2ZDT | 0.8524 | 0.8189 | 0.8365 | 0.9355 | 0.5007 | 104 | 23 |
| 2ETR | 0.7707 | 0.7826 | 0.81 | 0.931 | 0.3217 | 100 | 15 |
| 2OJ9 | 0.9048 | 0.8072 | 0.7703 | 0.9268 | 0.6177 | 148 | 75 |
| 2OI0 | 0.4277 | 0.4405 | 0.4436 | 0.9255 | -0.0776 | 532 | 31 |
| 1KVO | 0.5916 | 0.6106 | 0.6061 | 0.9231 | 0.1659 | 99 | 14 |
| 2GTK | 0.4812 | 0.8748 | 0.9483 | 0.918 | -0.0064 | 484 | 43 |
| 1BCD | 0.3076 | 0.4393 | 0.4512 | 0.9136 | -0.1453 | 492 | 27 |
| 2FSZ | 0.8414 | 0.7789 | 0.782 | 0.9082 | 0.5018 | 367 | 126 |
| 2ZEC | 0.5671 | 0.3659 | 0.3311 | 0.9074 | 0.0117 | 148 | 16 |
| 3G0E | 0.2492 | 0.4483 | 0.4699 | 0.907 | -0.2221 | 166 | 8 |
| 2AYW | 0.8323 | 0.8587 | 0.9198 | 0.9037 | 0.5584 | 449 | 117 |
| 3BGS | 0.5243 | 0.7257 | 0.7864 | 0.9 | -0.0801 | 103 | 10 |
| 3BZ3 | 0.7845 | 0.8739 | 0.97 | 0.8981 | -0.0553 | 100 | 11 |
| 3FRJ | 0.4385 | 0.4234 | 0.4212 | 0.8968 | -0.0718 | 330 | 29 |
| 2I78 | 0.8012 | 0.6757 | 0.651 | 0.8943 | 0.3477 | 533 | 167 |
| 2QD9 | 0.4768 | 0.6344 | 0.6696 | 0.8917 | 0.0172 | 578 | 73 |
| 3L5D | 0.6739 | 0.8148 | 0.8975 | 0.8912 | 0.1445 | 283 | 41 |
| 1ZW5 | 0.9089 | 0.9018 | 1 | 0.8854 | 0.7244 | 85 | 27 |
| 2ICA | 0.8126 | 0.7725 | 0.8333 | 0.8846 | 0.2883 | 138 | 29 |
| 3LQ8 | 0.5645 | 0.694 | 0.7651 | 0.8819 | -0.1665 | 166 | 17 |
| 3MAX | 0.6878 | 0.6397 | 0.6 | 0.881 | 0.3106 | 185 | 62 |
| 2V3F | 0.7665 | 0.6173 | 0.5 | 0.871 | 0.3413 | 54 | 27 |
| 1C8K | 0.2449 | 0.4167 | 0.4286 | 0.8684 | -0.1587 | 77 | 7 |
| 3BIZ | 0.7137 | 0.8462 | 0.9706 | 0.8684 | -0.0622 | 102 | 15 |
| 3D4Q | 0.6224 | 0.7389 | 0.8158 | 0.8671 | 0.1231 | 152 | 28 |
| 3F9M | 0.566 | 0.5377 | 0.5652 | 0.8525 | -0.0532 | 92 | 14 |
| 1NJS | 0.3917 | 0.8548 | 1 | 0.8475 | 0.4603 | 50 | 12 |
| 2P54 | 0.5887 | 0.8352 | 0.9839 | 0.8417 | 0.2737 | 373 | 82 |
| 1UDT | 0.5936 | 0.5906 | 0.6131 | 0.8385 | 0.0859 | 398 | 93 |
| 3CQW | 0.5117 | 0.4306 | 0.4061 | 0.838 | -0.0204 | 293 | 53 |
| 2NNQ | 0.8976 | 0.8413 | 0.9787 | 0.8364 | 0.5441 | 47 | 16 |
| 3D0E | 0.4909 | 0.7214 | 0.8376 | 0.8305 | -0.0325 | 117 | 23 |
| 2CNK | 0.4639 | 0.4534 | 0.4472 | 0.8241 | -0.0484 | 199 | 37 |
| 1SJ0 | 0.8385 | 0.79 | 0.9138 | 0.8216 | 0.4071 | 383 | 136 |
| 2HZI | 0.7618 | 0.7669 | 0.8516 | 0.8158 | 0.4476 | 182 | 84 |
| 1SQT | 0.6511 | 0.7282 | 0.8519 | 0.8118 | 0.1344 | 162 | 44 |
| 1H00 | 0.5763 | 0.618 | 0.6646 | 0.8098 | 0.1043 | 474 | 136 |
| 2E1W | 0.8169 | 0.7556 | 0.8495 | 0.8061 | 0.4121 | 93 | 42 |
| 3KL6 | 0.7082 | 0.7938 | 0.9572 | 0.8056 | 0.355 | 537 | 176 |
| 3CJO | 0.4587 | 0.6483 | 0.75 | 0.7982 | -0.008 | 116 | 29 |
| 1B9V | 0.3836 | 0.5714 | 0.6429 | 0.7975 | -0.0961 | 98 | 21 |
| 1W7X | 0.6327 | 0.6275 | 0.693 | 0.7822 | 0.1186 | 114 | 39 |
| 1UYG | 0.3191 | 0.7699 | 0.9773 | 0.7818 | 0.0446 | 88 | 25 |
| 2VT4 | 0.4913 | 0.4241 | 0.3725 | 0.7731 | -0.0161 | 247 | 69 |
| 1Q4X | 0.6538 | 0.6818 | 0.8447 | 0.7699 | -0.0612 | 103 | 29 |
| 1E66 | 0.8738 | 0.8074 | 0.8631 | 0.7667 | 0.6206 | 453 | 487 |
| 2ZNP | 0.6911 | 0.7586 | 0.9833 | 0.7638 | 0.1468 | 240 | 79 |
| 2OF2 | 0.5546 | 0.5863 | 0.6524 | 0.7548 | 0.0466 | 420 | 148 |
| 3EML | 0.5462 | 0.5994 | 0.6577 | 0.7512 | 0.1034 | 482 | 192 |
| 2OJG | 0.583 | 0.7281 | 0.9114 | 0.75 | 0.2855 | 79 | 35 |
| 3EL8 | 0.7293 | 0.7374 | 0.9256 | 0.736 | 0.3912 | 524 | 287 |
| 3F07 | 0.6409 | 0.7243 | 0.9588 | 0.7309 | 0.2284 | 170 | 73 |
| 3G6Z | 0.6223 | 0.7 | 0.9038 | 0.7287 | 0.19 | 104 | 46 |
| 2RGP | 0.7231 | 0.6617 | 0.7509 | 0.6863 | 0.3005 | 542 | 407 |
| 1YPE | 0.5304 | 0.5223 | 0.4881 | 0.6798 | 0.0695 | 461 | 255 |
| 2OWB | 0.5165 | 0.5948 | 0.8037 | 0.6772 | -0.1069 | 107 | 46 |
| 3NXO | 0.5906 | 0.658 | 0.9307 | 0.6761 | 0.0848 | 231 | 117 |
| 1SYN | 0.5376 | 0.6047 | 0.7248 | 0.6752 | 0.1256 | 109 | 63 |
| 3ODU | 0.325 | 0.4074 | 0.4 | 0.6667 | -0.1512 | 40 | 14 |
| 1QW6 | 0.6237 | 0.5491 | 0.49 | 0.6447 | 0.1195 | 100 | 73 |
| 3M2W | 0.7211 | 0.6593 | 0.8812 | 0.6403 | 0.3088 | 101 | 81 |
| 3NY8 | 0.4941 | 0.51 | 0.5974 | 0.6389 | -0.062 | 231 | 118 |
| 3LAN | 0.5733 | 0.6208 | 0.7959 | 0.6315 | 0.2056 | 338 | 258 |
| 2B8T | 0.5347 | 0.584 | 0.2281 | 0.619 | 0.1471 | 57 | 68 |
| 2I0E | 0.6744 | 0.6458 | 0.7111 | 0.6038 | 0.3004 | 135 | 153 |
| 1D3G | 0.6238 | 0.5729 | 0.7297 | 0.5956 | 0.1118 | 111 | 88 |
| 3E37 | 0.1515 | 0.2597 | 0.3091 | 0.5903 | -0.5095 | 592 | 132 |
| 2P2I | 0.4473 | 0.294 | 0.1663 | 0.5862 | -0.1843 | 409 | 142 |
| 3NXU | 0.6146 | 0.6407 | 0.2765 | 0.5802 | 0.1871 | 170 | 267 |
| 2AZR | 0.5894 | 0.5795 | 0.4692 | 0.5495 | 0.1454 | 130 | 153 |
| 1S3B | 0.5722 | 0.5538 | 0.5984 | 0.5368 | 0.1103 | 122 | 129 |
| 1J4H | 0.3829 | 0.3911 | 0.1802 | 0.5263 | -0.1003 | 111 | 68 |
| 2HV5 | 0.6234 | 0.3333 | 0.0755 | 0.5217 | -0.1041 | 159 | 78 |
| 3C4F | 0.5593 | 0.5228 | 0.6763 | 0.5081 | 0.0555 | 139 | 146 |
| 3LN1 | 0.5172 | 0.4972 | 0.7402 | 0.4946 | -0.0009 | 435 | 444 |
| 1R9O | 0.477 | 0.4696 | 0.3917 | 0.3588 | -0.0846 | 120 | 176 |
| 1VSO | 0.6121 | 0.4851 | 0.6634 | 0.3252 | 0.0677 | 101 | 235 |
| 3KGC | 0.2578 | 0.2953 | 0.2848 | 0.2432 | -0.4089 | 158 | 201 |
| 3NF7 | 0.4164 | 0.4728 | 0.39 | 0.2267 | -0.0948 | 100 | 268 |
| 2OYU | 0.3717 | 0.2617 | 0.6256 | 0.1241 | -0.1553 | 195 | 1070 |
| 2H7L | 0.1152 | 0.3385 | 0 | 0 | 0 | 43 | 22 |
| Average | 0.5907 | 0.6340 | 0.6745 | 0.7800 | 0.1203 | 225.3168 | 90.4455 |

**Table S2. The performance of Autodock Vina on the 20 selected protein targets with median score as cutoff.**

| Name | AUC | Accuracy | TPR | Precision | MCC | Cutoff  (kcal/mol) | Pos num | Neg num |
| --- | --- | --- | --- | --- | --- | --- | --- | --- |
| 3PBL | 0.6854 | 0.5234 | 0.5136 | 0.9919 | 0.1234 | -9.4 | 477 | 14 |
| 3KRJ | 0.6606 | 0.5294 | 0.5212 | 0.9885 | 0.1086 | -9.6 | 165 | 5 |
| 830C | 0.6228 | 0.5243 | 0.5149 | 0.9767 | 0.1003 | -9.6 | 571 | 26 |
| 1LRU | 0.4522 | 0.5182 | 0.5294 | 0.9153 | -0.0498 | -7.3 | 102 | 8 |
| 3CHP | 0.7953 | 0.6354 | 0.5906 | 1.0000 | 0.3692 | -10.4 | 171 | 21 |
| 2ZDT | 0.7569 | 0.6850 | 0.6442 | 0.9571 | 0.3978 | -9 | 104 | 23 |
| 2ETR | 0.3400 | 0.4435 | 0.4800 | 0.8000 | -0.2157 | -8.5 | 100 | 15 |
| 2OJ9 | 0.8216 | 0.7937 | 0.7365 | 0.9397 | 0.6082 | -8.8 | 148 | 75 |
| 2FSZ | 0.6498 | 0.6192 | 0.5852 | 0.8512 | 0.2639 | -8.9 | 352 | 126 |
| 2AYW | 0.6013 | 0.5676 | 0.5446 | 0.8622 | 0.1629 | -8 | 448 | 114 |
| 1ZW5 | 0.3893 | 0.4375 | 0.4824 | 0.6833 | -0.1898 | -6.6 | 85 | 27 |
| 2ICA | 0.5276 | 0.5337 | 0.5368 | 0.8488 | 0.0412 | -8.3 | 136 | 27 |
| 2NNQ | 0.7148 | 0.6667 | 0.6170 | 0.9063 | 0.3740 | -9.1 | 47 | 16 |
| 1SJ0 | 0.6620 | 0.6360 | 0.6055 | 0.8533 | 0.2879 | -9.1 | 365 | 135 |
| 2HZI | 0.6767 | 0.6541 | 0.6154 | 0.8358 | 0.3286 | -9.7 | 182 | 84 |
| 2E1W | 0.4270 | 0.4444 | 0.4731 | 0.6286 | -0.1352 | -7.8 | 93 | 42 |
| 3KL6 | 0.6807 | 0.6498 | 0.6201 | 0.8810 | 0.3115 | -9.5 | 537 | 174 |
| 1E66 | 0.6602 | 0.6591 | 0.6834 | 0.6303 | 0.3202 | -10 | 439 | 485 |
| 3EL8 | 0.6298 | 0.6275 | 0.6219 | 0.7570 | 0.2488 | -8.7 | 521 | 287 |
| Average | 0.6186 | 0.5868 | 0.5745 | 0.8583 | 0.1819 | -8.8579 | 265.4211 | 89.6842 |

Table S3. The performance of Schrödinger on the 5 selected protein targets with median score as cutoff. The docking was carried by Schrödinger’s HTVS mode, with the known ligand binding site as docking pocket.

| Name | AUC | Accuracy | TPR | Precision | MCC | Cutoff  (kcal/mol) | Pos num | Neg num |
| --- | --- | --- | --- | --- | --- | --- | --- | --- |
| 3PBL | 0.5247 | 0.5058 | 0.5040 | 0.9615 | 0.0200 | -4.9357 | 248 | 11 |
| 3KRJ | 0.6547 | 0.5181 | 0.5093 | 0.9880 | 0.1057 | -6.1231 | 161 | 5 |
| 830C | 0.5439 | 0.5078 | 0.5045 | 0.9655 | 0.0350 | -7.2146 | 555 | 24 |
| 1LRU | 0.5909 | 0.5238 | 0.5152 | 0.9623 | 0.0844 | -8.4945 | 99 | 6 |
| 3CHP | 0.7370 | 0.6159 | 0.5692 | 0.9737 | 0.3280 | -8.1263 | 130 | 21 |

**Table S4. The recall rate of top 10%, 20%, 30%, 40%, and 50%, respectively. The active compounds are mixed in the compound database.**

| **Name** | **active_num** | **total_num** | **RR_0.1** | **RR_0.2** | **RR_0.3** | **RR_0.4** | **RR_0.5** |
| --- | --- | --- | --- | --- | --- | --- | --- |
| 1NJS | 50 | 10402945 | 1 | 1 | 1 | 1 | 1 |
| 1ZW5 | 85 | 10402980 | 1 | 1 | 1 | 1 | 1 |
| 2AYW | 449 | 10403344 | 0.9599 | 0.9688 | 0.9733 | 0.9755 | 0.9866 |
| 2ZNP | 240 | 10403135 | 0.9375 | 0.975 | 0.9875 | 0.9917 | 1 |
| 3KL6 | 537 | 10403432 | 0.9143 | 0.9739 | 0.9944 | 1 | 1 |
| 3G6Z | 104 | 10402999 | 0.9135 | 0.9519 | 0.9615 | 0.9712 | 0.9712 |
| 1XL2 | 536 | 10403431 | 0.8787 | 0.9216 | 0.9515 | 0.959 | 0.9664 |
| 1UYG | 88 | 10402983 | 0.8636 | 0.9545 | 0.9773 | 1 | 1 |
| 2P54 | 373 | 10403268 | 0.8338 | 0.9517 | 0.9759 | 0.9893 | 0.992 |
| 3BIZ | 102 | 10402997 | 0.8137 | 0.9118 | 0.9314 | 0.9706 | 0.9902 |
| 1SJ0 | 383 | 10403278 | 0.812 | 0.8773 | 0.9269 | 0.9504 | 0.9713 |
| 3BZ3 | 100 | 10402995 | 0.81 | 0.94 | 0.95 | 0.97 | 1 |
| 1SQT | 162 | 10403057 | 0.7963 | 0.8889 | 0.9198 | 0.9321 | 0.9568 |
| 3CCW | 170 | 10403065 | 0.7824 | 0.8647 | 0.9176 | 0.9353 | 0.9588 |
| 3LQ8 | 166 | 10403061 | 0.7771 | 0.8193 | 0.8494 | 0.8855 | 0.9036 |
| 3E37 | 592 | 10403487 | 0.7686 | 0.875 | 0.9307 | 0.9696 | 0.9882 |
| 1B9V | 98 | 10402993 | 0.7653 | 0.8469 | 0.8878 | 0.949 | 0.9796 |
| 3D0E | 117 | 10403012 | 0.7436 | 0.8632 | 0.906 | 0.9231 | 0.9402 |
| 830C | 572 | 10403467 | 0.715 | 0.8497 | 0.9178 | 0.9633 | 0.9808 |
| 3F07 | 170 | 10403065 | 0.7118 | 0.9 | 0.9588 | 0.9588 | 0.9647 |
| 2ZEC | 148 | 10403043 | 0.7095 | 0.8446 | 0.8784 | 0.9257 | 0.9527 |
| 3EL8 | 524 | 10403419 | 0.6889 | 0.8702 | 0.9198 | 0.9466 | 0.9599 |
| 2E1W | 93 | 10402988 | 0.6882 | 0.7742 | 0.8925 | 0.9462 | 0.957 |
| 3L5D | 283 | 10403178 | 0.6855 | 0.788 | 0.8375 | 0.8905 | 0.9329 |
| 3HL5 | 100 | 10402995 | 0.68 | 0.83 | 0.89 | 0.92 | 0.94 |
| 2FSZ | 367 | 10403262 | 0.6703 | 0.7766 | 0.8229 | 0.8583 | 0.8747 |
| 1E66 | 453 | 10403348 | 0.6424 | 0.7506 | 0.819 | 0.8565 | 0.872 |
| 2HZI | 182 | 10403077 | 0.6319 | 0.8297 | 0.8901 | 0.9396 | 0.9725 |
| 1W7X | 114 | 10403009 | 0.6228 | 0.7105 | 0.7895 | 0.8509 | 0.9035 |
| 3EQH | 121 | 10403016 | 0.6198 | 0.7355 | 0.8595 | 0.9091 | 0.9256 |
| 2RGP | 542 | 10403437 | 0.6107 | 0.7362 | 0.7989 | 0.8598 | 0.8911 |
| 2I78 | 533 | 10403428 | 0.6041 | 0.7186 | 0.7955 | 0.8368 | 0.8593 |
| 3HMM | 133 | 10403028 | 0.594 | 0.9098 | 0.9624 | 0.9925 | 1 |
| 3BQD | 258 | 10403153 | 0.5775 | 0.7364 | 0.8527 | 0.9225 | 0.9767 |
| 1LRU | 102 | 10402997 | 0.5686 | 0.6471 | 0.6961 | 0.7353 | 0.8137 |
| 1SYN | 109 | 10403004 | 0.5596 | 0.6606 | 0.7064 | 0.7706 | 0.8073 |
| 3D4Q | 152 | 10403047 | 0.5592 | 0.7237 | 0.8355 | 0.8947 | 0.9342 |
| 2V3F | 54 | 10402949 | 0.537 | 0.537 | 0.5556 | 0.5556 | 0.5556 |
| 2NNQ | 47 | 10402942 | 0.5319 | 0.7872 | 0.8511 | 0.9149 | 0.9574 |
| 1YPE | 461 | 10403356 | 0.5206 | 0.6681 | 0.7354 | 0.8026 | 0.8482 |
| 1MV9 | 131 | 10403026 | 0.5038 | 0.6794 | 0.8244 | 0.9084 | 0.9695 |
| 3NXO | 231 | 10403126 | 0.4848 | 0.5584 | 0.6017 | 0.671 | 0.7316 |
| 3MAX | 185 | 10403080 | 0.4811 | 0.7135 | 0.7946 | 0.8486 | 0.9027 |
| 2ZDT | 104 | 10402999 | 0.4808 | 0.7308 | 0.8269 | 0.8558 | 0.9231 |
| 3CHP | 171 | 10403066 | 0.4795 | 0.6725 | 0.7953 | 0.8363 | 0.8772 |
| 3NXU | 170 | 10403065 | 0.4706 | 0.5941 | 0.6765 | 0.7529 | 0.7882 |
| 2AM9 | 269 | 10403164 | 0.4684 | 0.6691 | 0.7732 | 0.8439 | 0.8885 |
| 3F9M | 92 | 10402987 | 0.4674 | 0.6739 | 0.8152 | 0.9022 | 0.9239 |
| 1VSO | 101 | 10402996 | 0.4653 | 0.6139 | 0.6535 | 0.7624 | 0.802 |
| 1KVO | 99 | 10402994 | 0.4646 | 0.6768 | 0.8081 | 0.8788 | 0.9394 |
| 1Q4X | 103 | 10402998 | 0.4466 | 0.6408 | 0.7379 | 0.8447 | 0.9126 |
| 1BCD | 492 | 10403387 | 0.4085 | 0.6809 | 0.8272 | 0.876 | 0.8963 |
| 2AZR | 130 | 10403025 | 0.4077 | 0.6538 | 0.7077 | 0.7769 | 0.8077 |
| 1H00 | 474 | 10403369 | 0.4008 | 0.6013 | 0.7131 | 0.808 | 0.9135 |
| 3KRJ | 166 | 10403061 | 0.3976 | 0.6386 | 0.8373 | 0.8976 | 0.9217 |
| 3M2W | 101 | 10402996 | 0.396 | 0.7723 | 0.8515 | 0.8911 | 0.9208 |
| 3BGS | 103 | 10402998 | 0.3883 | 0.5922 | 0.7282 | 0.7864 | 0.8252 |
| 3PBL | 480 | 10403375 | 0.3875 | 0.6417 | 0.7708 | 0.8438 | 0.9083 |
| 2OJ9 | 148 | 10403043 | 0.3851 | 0.6419 | 0.7635 | 0.8311 | 0.8986 |
| 2ICA | 138 | 10403033 | 0.3841 | 0.5942 | 0.6667 | 0.7174 | 0.8043 |
| 3BKL | 282 | 10403177 | 0.3759 | 0.5142 | 0.6135 | 0.6738 | 0.7234 |
| 2ETR | 100 | 10402995 | 0.37 | 0.48 | 0.57 | 0.63 | 0.75 |
| 2OF2 | 420 | 10403315 | 0.369 | 0.5524 | 0.7071 | 0.8095 | 0.9167 |
| 3KBA | 293 | 10403188 | 0.3618 | 0.5358 | 0.6621 | 0.7611 | 0.843 |
| 2AA2 | 94 | 10402989 | 0.3511 | 0.5426 | 0.6702 | 0.7979 | 0.8936 |
| 2GTK | 484 | 10403379 | 0.3492 | 0.595 | 0.7748 | 0.8719 | 0.9215 |
| 3LPB | 107 | 10403002 | 0.3178 | 0.5327 | 0.7009 | 0.8224 | 0.9065 |
| 1LI4 | 63 | 10402958 | 0.3175 | 0.381 | 0.3968 | 0.5556 | 0.619 |
| 3NF7 | 100 | 10402995 | 0.31 | 0.46 | 0.58 | 0.66 | 0.71 |
| 3G0E | 166 | 10403061 | 0.3012 | 0.4699 | 0.5542 | 0.6205 | 0.6627 |
| 1L2S | 48 | 10402943 | 0.2917 | 0.3333 | 0.4792 | 0.5417 | 0.6667 |
| 3C4F | 139 | 10403034 | 0.2806 | 0.4388 | 0.5683 | 0.7122 | 0.8273 |
| 2OWB | 107 | 10403002 | 0.2617 | 0.486 | 0.7383 | 0.8411 | 0.8692 |
| 2OJG | 79 | 10402974 | 0.2405 | 0.5443 | 0.8101 | 0.9114 | 0.9241 |
| 3CJO | 116 | 10403011 | 0.2241 | 0.3017 | 0.4397 | 0.5086 | 0.5862 |
| 2CNK | 199 | 10403094 | 0.2211 | 0.3065 | 0.3618 | 0.4221 | 0.4523 |
| 3KGC | 158 | 10403053 | 0.2089 | 0.3038 | 0.443 | 0.4873 | 0.5759 |
| 2QD9 | 578 | 10403473 | 0.2042 | 0.3529 | 0.5138 | 0.6125 | 0.6886 |
| 1D3G | 111 | 10403006 | 0.1892 | 0.3243 | 0.4144 | 0.4324 | 0.4595 |
| 2OI0 | 532 | 10403427 | 0.1805 | 0.235 | 0.2951 | 0.3628 | 0.4154 |
| 1QW6 | 100 | 10402995 | 0.18 | 0.31 | 0.36 | 0.4 | 0.43 |
| 3LN1 | 435 | 10403330 | 0.1747 | 0.3241 | 0.446 | 0.5632 | 0.6644 |
| 1S3B | 122 | 10403017 | 0.1721 | 0.3033 | 0.4016 | 0.5082 | 0.5656 |
| 3LAN | 338 | 10403233 | 0.1686 | 0.3728 | 0.5562 | 0.6982 | 0.7663 |
| 2VT4 | 247 | 10403142 | 0.1579 | 0.2915 | 0.4777 | 0.6235 | 0.7409 |
| 3CQW | 293 | 10403188 | 0.1433 | 0.3993 | 0.5939 | 0.7167 | 0.8328 |
| 2OYU | 195 | 10403090 | 0.1385 | 0.2667 | 0.4 | 0.5077 | 0.5641 |
| 1UDT | 398 | 10403293 | 0.1256 | 0.1859 | 0.2688 | 0.3744 | 0.4447 |
| 2I0E | 135 | 10403030 | 0.1111 | 0.2963 | 0.4519 | 0.5852 | 0.7037 |
| 2P2I | 409 | 10403304 | 0.1002 | 0.2958 | 0.4694 | 0.643 | 0.7457 |
| 3NY8 | 231 | 10403126 | 0.0952 | 0.1861 | 0.2814 | 0.3853 | 0.5411 |
| 3L3M | 508 | 10403403 | 0.0787 | 0.1575 | 0.2421 | 0.3189 | 0.4252 |
| 3ODU | 40 | 10402935 | 0.075 | 0.175 | 0.25 | 0.35 | 0.425 |
| 3EML | 482 | 10403377 | 0.0747 | 0.1618 | 0.2573 | 0.3797 | 0.5187 |
| 1C8K | 77 | 10402972 | 0.0519 | 0.1429 | 0.3766 | 0.4675 | 0.5584 |
| 3FRJ | 330 | 10403225 | 0.0424 | 0.1061 | 0.2152 | 0.3273 | 0.4364 |
| 1R9O | 120 | 10403015 | 0.0417 | 0.1333 | 0.25 | 0.3167 | 0.45 |
| 2HV5 | 159 | 10403054 | 0.0189 | 0.0692 | 0.1509 | 0.2453 | 0.3082 |
| 1J4H | 111 | 10403006 | 0.018 | 0.036 | 0.1351 | 0.2342 | 0.3874 |
| 2B8T | 57 | 10402952 | 0.0175 | 0.0175 | 0.1228 | 0.2281 | 0.2632 |
| 2H7L | 43 | 10402938 | 0 | 0 | 0 | 0 | 0 |
| 3BWM | 41 | 10402936 | 0 | 0 | 0.0488 | 0.1463 | 0.3415 |
| Average | 225.7921 | 10506119.7525 | 0.4513 | 0.5874 | 0.6785 | 0.7447 | 0.7977 |

**Table S5. The prediction performance using score cutoff of 0.99 and 0.9, respectively. The ratio between recall rate of prediction and recall rate of random selection was also used here as an important performance indicator.**

| **Name** | **Total_num** | **Active_num** | **TPR_0.99** | **Random_0.99** | **TPR_0.9** | **Random_0.9** | **Ratio_0.99** | **Ratio_0.9** |
| --- | --- | --- | --- | --- | --- | --- | --- | --- |
| 3CCW | 10403065 | 170 | 0.3706 | 0.00003 | 0.5412 | 0.001 | 12353.3333 | 541.2 |
| 1XL2 | 10403431 | 536 | 0.4552 | 0.0001 | 0.6026 | 0.0007 | 4552.0000 | 860.8571 |
| 2AYW | 10403344 | 449 | 0.3252 | 0.0003 | 0.7528 | 0.0059 | 1084.0000 | 127.5932 |
| 1B9V | 10402993 | 98 | 0.2449 | 0.0003 | 0.449 | 0.0074 | 816.3333 | 60.6757 |
| 1ZW5 | 10402980 | 85 | 0.9647 | 0.0012 | 1 | 0.0369 | 803.9167 | 27.1003 |
| 3LQ8 | 10403061 | 166 | 0.488 | 0.0007 | 0.7349 | 0.0231 | 697.1429 | 31.8139 |
| 2ZEC | 10403043 | 148 | 0.0405 | 0.0001 | 0.1081 | 0.0013 | 405.0000 | 83.1538 |
| 2V3F | 10402949 | 54 | 0.1296 | 0.0004 | 0.2593 | 0.0041 | 324.0000 | 63.2439 |
| 1SYN | 10403004 | 109 | 0.3028 | 0.0022 | 0.5413 | 0.0919 | 137.6364 | 5.8901 |
| 3G6Z | 10402999 | 104 | 0.4615 | 0.0047 | 0.6154 | 0.0308 | 98.1915 | 19.9805 |
| 3KL6 | 10403432 | 537 | 0.5549 | 0.0069 | 0.8901 | 0.0631 | 80.4203 | 14.1062 |
| 1LI4 | 10402958 | 63 | 0.0317 | 0.0004 | 0.0952 | 0.009 | 79.2500 | 10.5778 |
| 3BKL | 10403177 | 282 | 0.0071 | 0.0001 | 0.039 | 0.0017 | 71.0000 | 22.9412 |
| 3E37 | 10403487 | 592 | 0.1301 | 0.0019 | 0.2078 | 0.0058 | 68.4737 | 35.8276 |
| 1SQT | 10403057 | 162 | 0.284 | 0.0042 | 0.6667 | 0.042 | 67.6190 | 15.8738 |
| 1NJS | 10402945 | 50 | 0.24 | 0.0049 | 1 | 0.0691 | 48.9796 | 14.4718 |
| 1LRU | 10402997 | 102 | 0.3824 | 0.0082 | 0.5686 | 0.1223 | 46.6341 | 4.6492 |
| 3F9M | 10402987 | 92 | 0.0217 | 0.0005 | 0.163 | 0.0166 | 43.4000 | 9.8193 |
| 3EQH | 10403016 | 121 | 0.405 | 0.01 | 0.5702 | 0.0699 | 40.5000 | 8.1574 |
| 2E1W | 10402988 | 93 | 0.5054 | 0.0144 | 0.6882 | 0.0838 | 35.0972 | 8.2124 |
| 3NXU | 10403065 | 170 | 0.0706 | 0.0023 | 0.1412 | 0.0113 | 30.6957 | 12.4956 |
| 1SJ0 | 10403278 | 383 | 0.5666 | 0.02 | 0.8068 | 0.0956 | 28.3300 | 8.4393 |
| 3F07 | 10403065 | 170 | 0.0706 | 0.0025 | 0.6882 | 0.0926 | 28.2400 | 7.4320 |
| 830C | 10403467 | 572 | 0.4353 | 0.0156 | 0.8217 | 0.1794 | 27.9038 | 4.5803 |
| 2I78 | 10403428 | 533 | 0.2176 | 0.0078 | 0.5084 | 0.0553 | 27.8974 | 9.1935 |
| 3KGC | 10403053 | 158 | 0.0633 | 0.0024 | 0.1392 | 0.0378 | 26.3750 | 3.6825 |
| 2FSZ | 10403262 | 367 | 0.3624 | 0.0162 | 0.6431 | 0.0829 | 22.3704 | 7.7575 |
| 1UYG | 10402983 | 88 | 0.6136 | 0.0326 | 0.9205 | 0.1187 | 18.8221 | 7.7548 |
| 2AZR | 10403025 | 130 | 0.0769 | 0.0041 | 0.2385 | 0.0377 | 18.7561 | 6.3263 |
| 1W7X | 10403009 | 114 | 0.193 | 0.011 | 0.5614 | 0.0702 | 17.5455 | 7.9972 |
| 1VSO | 10402996 | 101 | 0.1386 | 0.0079 | 0.4554 | 0.0832 | 17.5443 | 5.4736 |
| 1YPE | 10403356 | 461 | 0.0716 | 0.0042 | 0.2538 | 0.0258 | 17.0476 | 9.8372 |
| 1MV9 | 10403026 | 131 | 0.1221 | 0.0074 | 0.4885 | 0.0971 | 16.5000 | 5.0309 |
| 3BQD | 10403153 | 258 | 0.3411 | 0.022 | 0.6512 | 0.1435 | 15.5045 | 4.5380 |
| 2RGP | 10403437 | 542 | 0.048 | 0.0031 | 0.5406 | 0.0743 | 15.4839 | 7.2759 |
| 2HZI | 10403077 | 182 | 0.1154 | 0.0076 | 0.6264 | 0.098 | 15.1842 | 6.3918 |
| 2ZNP | 10403135 | 240 | 0.8375 | 0.056 | 0.9625 | 0.1393 | 14.9554 | 6.9095 |
| 3BIZ | 10402997 | 102 | 0.3333 | 0.0223 | 0.902 | 0.1881 | 14.9462 | 4.7953 |
| 3BZ3 | 10402995 | 100 | 0.49 | 0.0337 | 0.91 | 0.1633 | 14.5401 | 5.5726 |
| 3D4Q | 10403047 | 152 | 0.1513 | 0.0125 | 0.5987 | 0.1173 | 12.1040 | 5.1040 |
| 3L5D | 10403178 | 283 | 0.5795 | 0.0519 | 0.8092 | 0.2325 | 11.1657 | 3.4804 |
| 1KVO | 10402994 | 99 | 0.0303 | 0.0029 | 0.2424 | 0.0473 | 10.4483 | 5.1247 |
| 3KRJ | 10403061 | 166 | 0.0904 | 0.0091 | 0.5241 | 0.138 | 9.9341 | 3.7978 |
| 2AA2 | 10402989 | 94 | 0.2128 | 0.0221 | 0.5 | 0.1697 | 9.6290 | 2.9464 |
| 3BGS | 10402998 | 103 | 0.2136 | 0.0223 | 0.5243 | 0.1587 | 9.5785 | 3.3037 |
| 2P54 | 10403268 | 373 | 0.7989 | 0.0851 | 0.9625 | 0.2288 | 9.3878 | 4.2067 |
| 1Q4X | 10402998 | 103 | 0.2621 | 0.0297 | 0.6019 | 0.1912 | 8.8249 | 3.1480 |
| 3EL8 | 10403419 | 524 | 0.1508 | 0.0172 | 0.8244 | 0.1569 | 8.7674 | 5.2543 |
| 2AM9 | 10403164 | 269 | 0.0706 | 0.0098 | 0.5539 | 0.1354 | 7.2041 | 4.0908 |
| 3HL5 | 10402995 | 100 | 0.53 | 0.0754 | 0.84 | 0.2151 | 7.0292 | 3.9052 |
| 2OJ9 | 10403043 | 148 | 0.0405 | 0.006 | 0.3851 | 0.102 | 6.7500 | 3.7755 |
| 1E66 | 10403348 | 453 | 0.6424 | 0.0988 | 0.7947 | 0.2594 | 6.5020 | 3.0636 |
| 3NXO | 10403126 | 231 | 0.4416 | 0.0717 | 0.671 | 0.4033 | 6.1590 | 1.6638 |
| 2ZDT | 10402999 | 104 | 0.2404 | 0.0407 | 0.7019 | 0.1622 | 5.9066 | 4.3274 |
| 1BCD | 10403387 | 492 | 0.0549 | 0.0093 | 0.2724 | 0.0544 | 5.9032 | 5.0074 |
| 2ETR | 10402995 | 100 | 0.11 | 0.0214 | 0.52 | 0.254 | 5.1402 | 2.0472 |
| 1H00 | 10403369 | 474 | 0.2405 | 0.0515 | 0.5084 | 0.1416 | 4.6699 | 3.5904 |
| 2ICA | 10403033 | 138 | 0.3406 | 0.0777 | 0.6594 | 0.2905 | 4.3835 | 2.2699 |
| 2NNQ | 10402942 | 47 | 0.7872 | 0.1832 | 0.9362 | 0.4683 | 4.2969 | 1.9991 |
| 3KBA | 10403188 | 293 | 0.0887 | 0.0209 | 0.4403 | 0.1396 | 4.2440 | 3.1540 |
| 2OWB | 10403002 | 107 | 0.028 | 0.0066 | 0.2991 | 0.1148 | 4.2424 | 2.6054 |
| 2OF2 | 10403315 | 420 | 0.05 | 0.0118 | 0.3857 | 0.1092 | 4.2373 | 3.5321 |
| 3C4F | 10403034 | 139 | 0.2158 | 0.0547 | 0.4388 | 0.2066 | 3.9452 | 2.1239 |
| 1L2S | 10402943 | 48 | 0.125 | 0.0322 | 0.3333 | 0.2303 | 3.8820 | 1.4472 |
| 3LPB | 10403002 | 107 | 0.1682 | 0.0459 | 0.5701 | 0.2177 | 3.6645 | 2.6187 |
| 3CHP | 10403066 | 171 | 0.0409 | 0.0112 | 0.614 | 0.18 | 3.6518 | 3.4111 |
| 3PBL | 10403375 | 480 | 0.4396 | 0.1207 | 0.775 | 0.3029 | 3.6421 | 2.5586 |
| 3M2W | 10402996 | 101 | 0.1782 | 0.0496 | 0.6832 | 0.1722 | 3.5927 | 3.9675 |
| 2GTK | 10403379 | 484 | 0.3843 | 0.1166 | 0.8388 | 0.3555 | 3.2959 | 2.3595 |
| 3HMM | 10403028 | 133 | 0.1053 | 0.0325 | 0.8496 | 0.1688 | 3.2400 | 5.0332 |
| 2OI0 | 10403427 | 532 | 0.1203 | 0.0412 | 0.2801 | 0.2764 | 2.9199 | 1.0134 |
| 3CJO | 10403011 | 116 | 0.1121 | 0.0392 | 0.4397 | 0.3027 | 2.8597 | 1.4526 |
| 3MAX | 10403080 | 185 | 0.0054 | 0.002 | 0.2811 | 0.0382 | 2.7000 | 7.3586 |
| 2OJG | 10402974 | 79 | 0.1519 | 0.0583 | 0.6203 | 0.2268 | 2.6055 | 2.7350 |
| 3LAN | 10403233 | 338 | 0.068 | 0.0263 | 0.5148 | 0.2796 | 2.5856 | 1.8412 |
| 1D3G | 10403006 | 111 | 0.1441 | 0.0776 | 0.4324 | 0.4157 | 1.8570 | 1.0402 |
| 3G0E | 10403061 | 166 | 0.006 | 0.0033 | 0.2289 | 0.0661 | 1.8182 | 3.4629 |
| 1QW6 | 10402995 | 100 | 0.07 | 0.0388 | 0.36 | 0.3039 | 1.8041 | 1.1846 |
| 1S3B | 10403017 | 122 | 0.1803 | 0.1052 | 0.4016 | 0.3039 | 1.7139 | 1.3215 |
| 3LN1 | 10403330 | 435 | 0.0805 | 0.0492 | 0.4506 | 0.3013 | 1.6362 | 1.4955 |
| 2QD9 | 10403473 | 578 | 0.0069 | 0.0045 | 0.2976 | 0.1578 | 1.5333 | 1.8859 |
| 2VT4 | 10403142 | 247 | 0.0648 | 0.0459 | 0.1903 | 0.1377 | 1.4118 | 1.3820 |
| 2OYU | 10403090 | 195 | 0.1179 | 0.0858 | 0.4359 | 0.3296 | 1.3741 | 1.3225 |
| 1C8K | 10402972 | 77 | 0.013 | 0.0097 | 0.0649 | 0.1209 | 1.3402 | 0.5368 |
| 1UDT | 10403293 | 398 | 0.1131 | 0.0864 | 0.407 | 0.4327 | 1.3090 | 0.9406 |
| 3NY8 | 10403126 | 231 | 0.1558 | 0.1594 | 0.3506 | 0.3806 | 0.9774 | 0.9212 |
| 3EML | 10403377 | 482 | 0.0353 | 0.0479 | 0.2822 | 0.324 | 0.7370 | 0.8710 |
| 2I0E | 10403030 | 135 | 0.0593 | 0.0869 | 0.4667 | 0.3107 | 0.6824 | 1.5021 |
| 1R9O | 10403015 | 120 | 0.0167 | 0.0246 | 0.1167 | 0.1781 | 0.6789 | 0.6552 |
| 3FRJ | 10403225 | 330 | 0.0242 | 0.0865 | 0.1939 | 0.2844 | 0.2798 | 0.6818 |
| 3L3M | 10403403 | 508 | 0.002 | 0.008 | 0.1752 | 0.2201 | 0.2500 | 0.7960 |
| 1J4H | 10403006 | 111 | 0 | 0.0143 | 0.027 | 0.1419 | 0.0000 | 0.1903 |
| 2B8T | 10402952 | 57 | 0 | 0.0265 | 0.0175 | 0.1698 | 0.0000 | 0.1031 |
| 2CNK | 10403094 | 199 | 0 | 0.0137 | 0.2663 | 0.1427 | 0.0000 | 1.8662 |
| 2H7L | 10402938 | 43 | 0 | 0.0042 | 0 | 0.0316 | 0.0000 | 0.0000 |
| 2HV5 | 10403054 | 159 | 0 | 0.0028 | 0.0063 | 0.0505 | 0.0000 | 0.1248 |
| 2P2I | 10403304 | 409 | 0 | 0.0142 | 0.0489 | 0.0594 | 0.0000 | 0.8232 |
| 3BWM | 10402936 | 41 | 0 | 0.0177 | 0 | 0.1413 | 0.0000 | 0.0000 |
| 3CQW | 10403188 | 293 | 0 | 0.0074 | 0.0819 | 0.0696 | 0.0000 | 1.1767 |
| 3D0E | 10403012 | 117 | 0 | 0.0009 | 0.4188 | 0.0304 | 0.0000 | 13.7763 |
| 3NF7 | 10402995 | 100 | 0 | 0.004 | 0.17 | 0.0397 | 0.0000 | 4.2821 |
| 3ODU | 10402935 | 40 | 0 | 0.0158 | 0.1 | 0.1665 | 0.0000 | 0.6006 |
| Average | 10403119 | 223.5784 | 0.2046 | 0.0289 | 0.4759 | 0.1474 | 220.2948 | 22.1558 |

**Table S6. The top 12 poor performance cases (Ratio_0.99<0.25, ratio_0.9<1.5).**

| Gene name | Protein name | PDBID | Ratio_0.99 | ratio_0.9 | Protein and property |
| --- | --- | --- | --- | --- | --- |
| ADRB2 | Beta-2 adrenergic receptor | 3NY8 | 0.9774 | 0.9212 | Membrane protein |
| AA2AR | Adenosine A2a receptor | 3EML | 0.7370 | 0.8710 | Membrane protein |
| CP2C9 | Cytochrome P450 2C9 | 1R9O | 0.6789 | 0.6552 | The Pocket is buried inside of the protein and relative small,ligand contain ions |
| DHI1 | 11-beta-hydroxysteroid dehydrogenase 1 | 3FRJ | 0.2798 | 0.6818 | Two ligand in on pocket |
| PARP1 | Poly [ADP-ribose] polymerase-1 | 3L3M | 0.2500 | 0.7960 | The pocket is relative empty，ligand is largely expose to solvent |
| FKB1A | FK506-binding protein 1A | 1J4H | 0 | 0.1903 | The pocket is relative small， the ligand is largely expose to solvent |
| KITH | Thymidine kinase | 2B8T | 0 | 0.1031 | The pocket is inside of the protein and relative small |
| INHA | Enoyl-[acyl-carrier-protein] reductase | 2H7L | 0 | 0 | Two ligand in on pocket |
| ALDR | Aldose reductase | 2HV5 | 0 | 0.1248 | Contain other ligand NAP in the pocket |
| VGFR2 | Vascular endothelial growth factor receptor 2 | 2P2I | 0 | 0.8232 | The pocket is inside of the protein and relative small |
| COMT | Catechol O-methyltransferase | 3BWM | 0 | 0 | More than two ligands in the same pocket,and have ions in the pocket |
| CXCR4 | C-X-C chemokine receptor type 4 | 3ODU | 0 | 0.6006 | Membrane protein; multiply pocket |

**Table S7. The candidate list for experimental validation.**

| ZINCID | Commercial ID | DFCNN score | Vina Docking score（Kcal/mol） |
| --- | --- | --- | --- |
| ZINC000009426598 | STK260654# | 0.9941 | -10.2 |
| ZINC000257180903 | S763-0509 | 0.9965 | -10 |
| ZINC000021911270 | PB90939671 | 0.9930 | -9.6 |
| ZINC000003337861 | Z25746562# | 0.9939 | -9.2 |
| ZINC000016034019 | STK573808* | 0.9905 | -9.2 |
| ZINC000033249573 | E638-0079# | 0.9930 | -9.2 |
| ZINC000257195066 | S731-0210 | 0.9932 | -9.2 |
| ZINC000070005414 | Z850606558# | 0.9985 | -8 |
| ZINC000035966496 | V005-8078# | 0.9991 | -7.3 |

In column 2, # stands for Racemate, and * stands for Isomer, others are Exact.

**Table S8. Binding variables for compounds binding to trypsin.**

| Compound name | Ic 50 (mg/mL) | K_a_ (L mol^-1^) | n |
| --- | --- | --- | --- |
| S763-0509 | 5.21 | 6.77× 10^3^ | 1.17 |
| PB90939671 | 1.38 | 1.87× 10^3^ | 0.89 |
| STK573808 | 1.16 | 1.86× 10^6^ | 1.54 |
| STK260654 | 1.42 | 3.60× 10^4^ | 1.25 |
| Z25746562 | 2.98 | 1.39× 10^6^ | 1.49 |

**Reference**

Darden, T., York, D., and Pedersen, L. (1993). Particle mesh Ewald: An N ⋅log( N ) method for Ewald sums in large systems. *J. Chem. Phys.* 98, 10089–10092. doi:10.1063/1.464397.

Feng, Y., Lv, M., Lu, Y. Q., Liu, K., Liu, L., He, Z., et al. (2018). Characterization of binding interactions between selected phenylpropanoid glycosides and trypsin. *Food Chem.* doi:10.1016/j.foodchem.2017.09.118.

Hess, B., Kutzner, C., and Spoel, D. Van Der (2008). GROMACS 4: algorithms for highly efficient, load-balanced, and scalable molecular simulation. *J. Chem.*

Hornak, V., and Simmerling, C. (2003). Generation of accurate protein loop conformations through low-barrier molecular dynamics. *Proteins Struct. Funct. Genet.* doi:10.1002/prot.10363.

Humphrey, W., Dalke, A., and Schulten, K. (1996). VMD: visual molecular dynamics. *J. Mol. Graph.* 14, 33–8, 27–8. doi:10.1016/0263-7855(96)00018-5.

Jorgensen, W. L., Chandrasekhar, J., Madura, J. D., Impey, R. W., and Klein, M. L. (1983). Comparison of simple potential functions for simulating liquid water. *J. Chem. Phys.* 79, 926–935. doi:10.1063/1.445869.

Landrum, G. (2006). RDKit: Open-source Cheminformatics. *Http://Www.Rdkit.Org/*. doi:10.2307/3592822.

Murtagh, F., and Contreras, P. (2012). Algorithms for hierarchical clustering: An overview. *Wiley Interdiscip. Rev. Data Min. Knowl. Discov.* doi:10.1002/widm.53.

Sousa Da Silva, A. W., and Vranken, W. F. (2012). ACPYPE - AnteChamber PYthon Parser interfacE. *BMC Res. Notes*. doi:10.1186/1756-0500-5-367.

Wang, J., Wang, W., Kollman, P. A., and Case, D. A. (2006). Automatic atom type and bond type perception in molecular mechanical calculations. *J. Mol. Graph. Model.* 25, 247–260. doi:10.1016/j.jmgm.2005.12.005.

Xu, H., Lu, Y., Zhang, T., Liu, K., Liu, L., He, Z., et al. (2019). Characterization of binding interactions of anthraquinones and bovine β-lactoglobulin. *Food Chem.* 281, 28–35. doi:10.1016/j.foodchem.2018.12.077.

Zhang, H., Yang, Y., Li, J., Wang, M., Saravanan, K. M., Wei, J., et al. (2020). A novel virtual screening procedure identifies Pralatrexate as inhibitor of SARS-CoV-2 RdRp and it reduces viral replication in vitro. *PLoS Comput. Biol.* 16, e1008489. doi:10.1371/journal.pcbi.1008489.
